# Alterations in Cell Motility, Proliferation, and Metabolism in Novel Models of Acquired Temozolomide Resistant Glioblastoma

**DOI:** 10.1038/s41598-018-25588-1

**Published:** 2018-05-08

**Authors:** D. M. Tiek, J. D. Rone, G. T. Graham, E. L. Pannkuk, B. R. Haddad, R. B. Riggins

**Affiliations:** Department of Oncology, Lombardi Comprehensive Cancer Center, Georgetown University Medical Center, Washington, DC USA

## Abstract

Glioblastoma (GBM) is an aggressive and incurable tumor of the brain with limited treatment options. Current first-line standard of care is the DNA alkylating agent temozolomide (TMZ), but this treatment strategy adds only ~4 months to median survival due to the rapid development of resistance. While some mechanisms of TMZ resistance have been identified, they are not fully understood. There are few effective strategies to manage therapy resistant GBM, and we lack diverse preclinical models of acquired TMZ resistance in which to test therapeutic strategies on TMZ resistant GBM. In this study, we create and characterize two new GBM cell lines resistant to TMZ *in vitro*, based on the 8MGBA and 42MGBA cell lines. Analysis of the TMZ resistant (TMZres) variants in conjunction with their parental, sensitive cell lines shows that acquisition of TMZ resistance is accompanied by broad phenotypic changes, including increased proliferation, migration, chromosomal aberrations, and secretion of cytosolic lipids. Importantly, each TMZ resistant model captures a different facet of the “go” (8MGBA-TMZres) or “grow” (42MGBA-TMZres) hypothesis of GBM behavior. These *in vitro* model systems will be important additions to the available tools for investigators seeking to define molecular mechanisms of acquired TMZ resistance.

## Introduction

Glioblastoma (GBM) is the most common glioma among adults and confers an abysmally low overall survival with only 5% of patients surviving at the 5-year mark^1^. Over the past 33 years – 1980–2013 – 570 clinical trials were conducted where almost 33,000 patients were treated with different novel therapeutics to better understand and treat GBM^2^. From these extensive studies one chemotherapeutic agent – temozolomide (TMZ) – was found to moderately improve overall survival^3^. In the last decade there has been little advancement in treatment, with the standard of care being radiotherapy and surgery, followed by TMZ^4^. However, resistance to TMZ is rapid, and a broadly effective second line of treatment has not yet been established^5^. For these reasons, we need better models to understand mechanisms of TMZ resistance and how to develop improved therapies for the future.

Cell line models have been invaluable in elucidating the molecular mechanisms behind the uncontrolled growth of cancer cells. As resistance to TMZ is rapid in clinical models, cell lines were used to better understand the mechanism behind the initial efficacy of TMZ sensitivity. TMZ is a prodrug that is preferentially activated in a more alkaline environment, which the brain provides, that spontaneously breaks down to highly reactive methyldiazonium cations. These byproducts preferentially methylate DNA bases at the *N7*-guanine, *N3-*adenine, and *O6*-guanine positions. The cytotoxic effect was shown to be mainly through the *O6*-guanine adduct, which can be reversed by the O6-methylguanine methyl transferase (MGMT). MGMT is a suicide repair protein which removes the *O6-*guanine adduct and allows for proper DNA repair^6^. Even with this information, targeting MGMT with both small molecule drugs and mimetics has been unsuccessful, and its expression does not always correlate with resistance to TMZ^7–11^. Therefore, new models of resistance need to be developed in order to better define molecular mechanisms of GBM resistance to TMZ.

In this study, we present two unique TMZ-resistant GBM cell lines derived from the 42MGBA and 8MGBA parental lines which were first characterized in 1997. The 42MGBA line was derived from a temporal lobe tumor resected from a 63-year old male. The 8MGBA line was derived from a frontal lobe tumor resected from a 54-year old female^12^. After continual exposure to TMZ in culture, both cell lines no longer undergo TMZ-mediated G2/M arrest. As the cell lines acquired resistance to TMZ both cell lines gained expression of MGMT, showed an increase in nuclear size and chromosome number, and an increase of early endosomes and extracellular lipids. However, as they differed in their growth and migratory capacity, these cell lines may give us insight into the “go or grow” model for GBM recurrence^13^. The 42MGBA-TMZ resistant cell line had an increase in growth capturing the “go” of the “go or grow” model for resistance. By contrast, the 8MBGA-TMZ resistant cell line had an increase in migration, encompassing the “grow” aspect of resistance. These models will be useful tools for the field to better define essential drug resistance mechanisms in GBM. These two TMZ-resistant (TMZres) models from both a male and female patient have evolved distinct resistant phenotypes and therefore serve as valuable resources to investigate the heterogeneity of TMZ-resistant GBM.

## Results

### Acquired temozolomide resistance decreases sensitivity to BCNU

**Figure 1:**
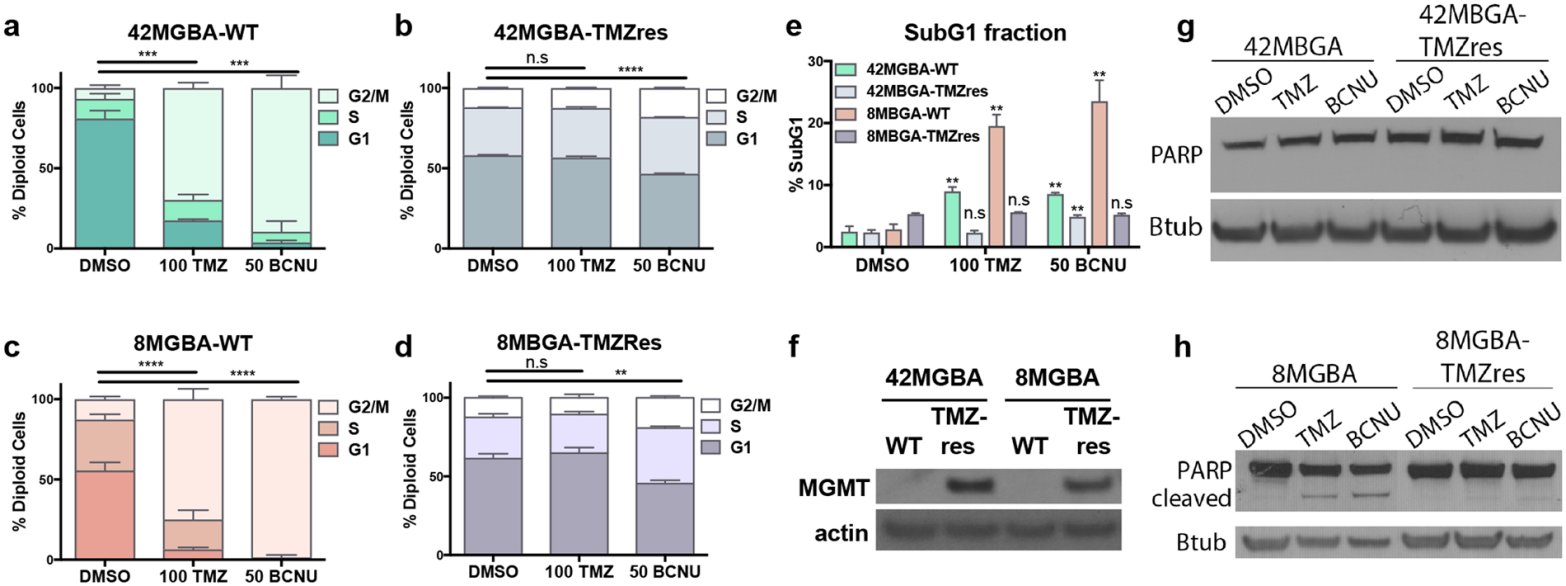
Acquired TMZ resistance. (**a**,**c**) Cell cycle analysis of the parental 42MGBA (**a**) and 8MGBA cell lines (**c**) with 100 μM TMZ and 50 μM BCNU treatment for 72 hr. One-way ANOVA p = 0.0003; p = <0.0001 (**b,d**) Cell cycle analysis of 42MGBA- **(b**) and 8MBGA-TMZres cell lines (**d**) with 100 μM TMZ and 50 μM BCNU treatment for 72 hours. One-way ANOVA p = <0.0001; p = 0.0010. (**e**) Cell cycle analysis of SubG1 fraction for parental and resistant cell lines. t-test of treatment vs DMSO 42MGBA-WT p = 0.0046, 42MBGA-TMZres p = 0.0065, 8MBGA p = 0.0011. (**f**) TMZres cells express MGMT. (**g**,**h**) TMZ and BCNU induce PARP cleavage in 8MGBA-WT cells, but not 8MGBA-TMZres, 42MGBA-WT, or 42MGBA-TMZres cells. Cells were treated for 72 hours prior to lysis and western blot analysis.

To better recapitulate acute temozolomide (TMZ) resistance models, we created cell lines that were resistant to 200 µM TMZ and were challenged in 100 µM TMZ for all experiments. The overall time to acquired resistance varied from approximately 2 months for the 8MBGA cell line to ~3 months for the 42MBGA cell line. Cells were defined as resistant when each no longer showed a G2/M arrest or apoptosis in response to TMZ treatment (Fig. 1a–d). While both parental cell lines (WT) exhibited a significant increase in the SubG1, or apoptotic, fraction following TMZ treatment, this was lost in the TMZres variants (Fig. 1e). However, the shape of the SubG1 curves differed between the two cell lines – 8MGBA-WT cells treated with TMZ showed a defined SubG1 peak typically associated with apoptosis, while 42MGBA-WT cells did not (Supp Fig. 1a,b). This was corroborated by immunoblotting for cleaved poly (ADP-ribose) polymerase (PARP), which was observed in 8MGBA-WT but not 42MGBA-WT cells (Fig. 1g,h). Expression of the O6-methylguanine methyltransferase (MGMT) was detectable in both resistant cell lines, but absent in both parental lines (Fig. 1f).

Acquired resistance to the previous standard of care for GBM, bis-chloroethylnitrosourea (BCNU), leads to an increased sensitivity to TMZ^14^. Because BCNU can still be a component of second-line treatment for GBM^15–18^, we tested whether our TMZres cells exhibited altered responsiveness to BCNU. Since the resistant cell lines are populations selected from the wild type cell lines they have a more reproducible phenotype as the variability or heterogeneity of the cell line has decreased. Therefore, the TMZres cells still showed a statistically significant G2/M arrest upon BCNU treatment. However the biological effect is dramatically reduced, with a 74% increase in G2/M vs 8% increase in G2/M for the 42MBGA-WT vs. 42MBGA-TMZres cells compared to controls, respectively (Fig. 1a,b). This is similar to 8MGBA-WT and 8MGBA-TMZres cells, where the increase in G2/M fraction is 86% vs. 7% compared to controls, respectively (Fig. 1c,d).

### Increase in nuclear size and chromosome number with TMZ resistance

**Figure 2:**
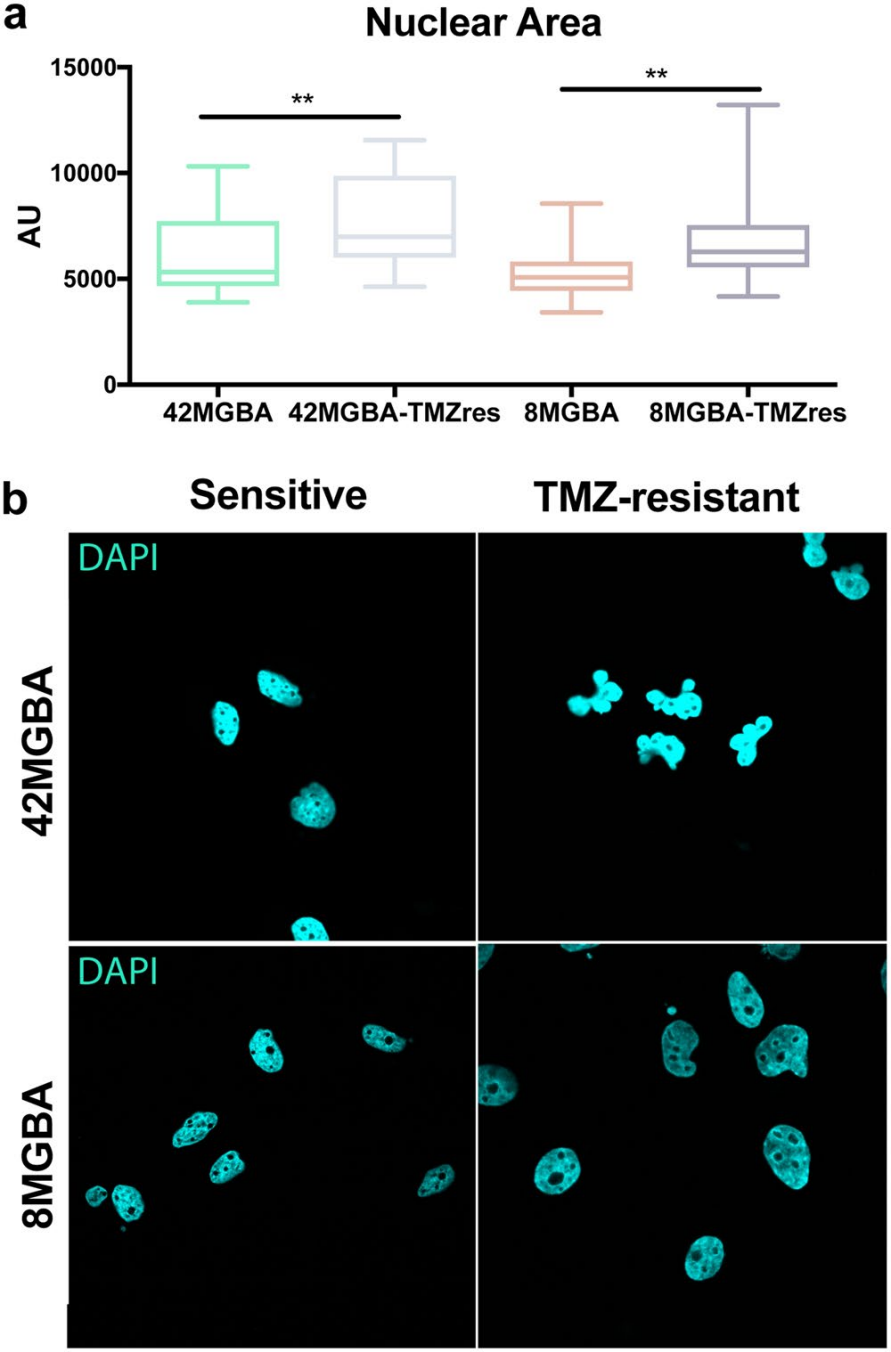
Changes in nuclear size and shape upon acquired TMZ resistance. (**a**) Quantification of nuclear area by pixel size. AU = arbitrary units. Two-tailed t-test 42MBGA p = 0.0035; 8MBGA p = 0.0034. (**b**) Representative image of nuclear size, quantified in a. Change in nuclear structure in 42MGBA-TMZres in comparison to 42MBGA-WT.

The mechanism of action for TMZ is to induce DNA damage and give rise to cell cycle arrest in the G2/M phase^5^. As cells have already copied their DNA at this point in the cell cycle, if a cell is to survive TMZ-treatment they may also be retaining the extra copies of chromosomes that have already been duplicated. The 42MGBA-WT cell line has a hypertetraploid karyotype (88–95 chromosomes) with 8% polyploidy, while the 8MGBA-WT cell line has a hyperdiploid karyotype with 15% polyploidy and contains 47–52 chromosomes (DSMZ, https://www.dsmz.de/). 42MGBA-TMZres cells showed a significant increase in total nuclear area (Fig. 2a), and their nuclear morphology became multi-lobed (Fig. 2b). Total nuclear area was also significantly increased in 8MGBA-TMZres cells, though their nuclei retained an oblong or circular morphology (Fig. 2a,b). Given the strikingly different nuclear morphology of the two resistant variants, we cultured both TMZres lines in the absence of TMZ for three weeks to test the stability of the resistant phenotype. After re-challenging with TMZ treatment for 72 hours, we observed no significant change in cell cycle profile or the SubG1 peak in the 42MBGA-TMZres cell line, while the 8MBGA-TMZres line underwent a slight G2/M arrest and showed a unique SubG1 peak in both untreated and treated conditions suggesting a less stable phenotype (Supp Fig. 2a–d).

**Figure 3:**
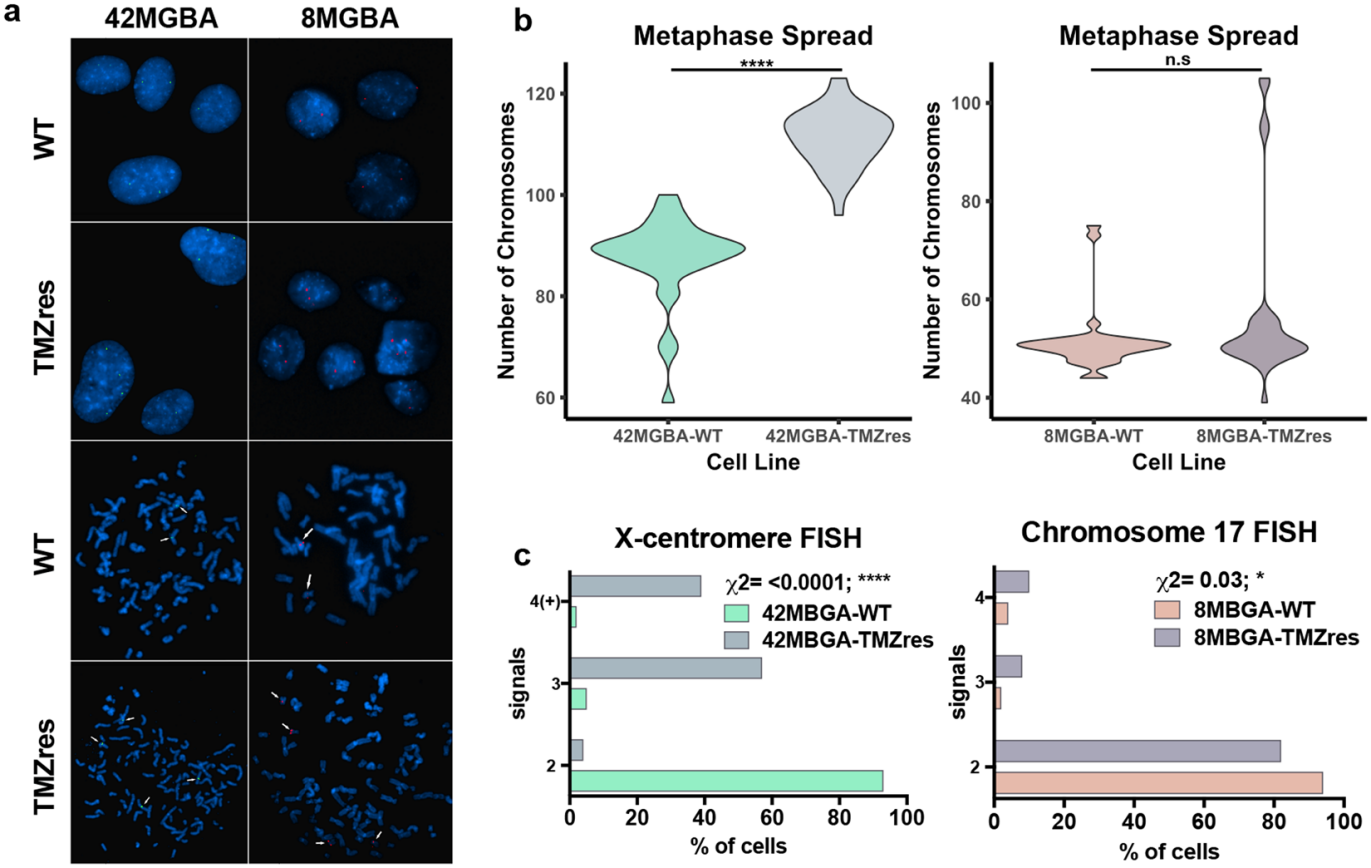
Acquired TMZ resistance is associated with chromosomal copy number increase. (**a**) Bottom 4 panels: metaphase spreads from TMZres cells showing overall chromosomal copy number gain compared to parental cells, and multiple copies of chromosomes 17 (8MGBA-TMZres, red signal, arrows) and X (42MGBA-TMZres, green signal, arrows). Metaphase spreads from the parental cells show 2 copies of the respective chromosomes. Top 4 panels: interphase nuclei from TMZres cells showing multiple copies of chromosomes 17 (8MGBA-TMZres, red signal) and X (42MGBA-TMZres cells, green signal) and two copies in the respective parental cells. (**b**) Quantification of chromosomes from a, bottom 4 panels 42MGBA-WT vs –TMZres p = <0.0001. (**c**) Quantification of probe signal from a, top 4 panels. Chi-squared test 8MBGA p = 0.03; 42MBGA p = <0.0001.

To determine whether the increase in the total nuclear area in TMZ-resistant cells was associated with chromosomal gain, we assessed the overall chromosomal number in metaphase spreads from the TMZres cell lines compared to their respective parental lines (WT). We observed an increase in overall chromosome number in the majority of the 42MBGA-TMZres cells in contrast to the 8MBGA-TMZres cells where only a small subpopulation of cells had an increase in overall chromosome number (Fig. 3b). We also assessed the change in copy number of a representative chromosome in each cell line pair (chromosome 17 in the 8MGBA pair and X in the 42MGBA pair), in interphase nuclei and metaphase spreads (200/cell line) using fluorescent *in situ* hybridization (FISH) (Fig. 3a). The choice of the two representative chromosomes was made based on reported karyotype analysis of the 2 parental cell lines showing a mostly diploid count for chromosome 17 in the 8MGBA line and X in 42MGBA (DSMZ, https://www.dsmz.de/). We observed that 96% of the 42MBGA-TMZres cells had three or more copies of the X chromosome compared to only 7% of the 42MBGA-WT cells (93% of those cells had 2 copies). In contrast, this dramatic shift was not observed in 8MBGA-TMZres cells, where only a small subpopulation of cells showed an increase in the number of chromosomes 17 (18% had 3 or more copies) compared to the parental cells (6% had 3 or more copies). Taken together, these findings tracked with the stability of TMZ-resistance, with the 42MBGA-TMZres cells showing a more stable phenotype compared to 8MBGA-TMZres cells (Fig. 3c).

### Changes in proliferation, migration, and actin cytoskeleton

**Figure 4:**
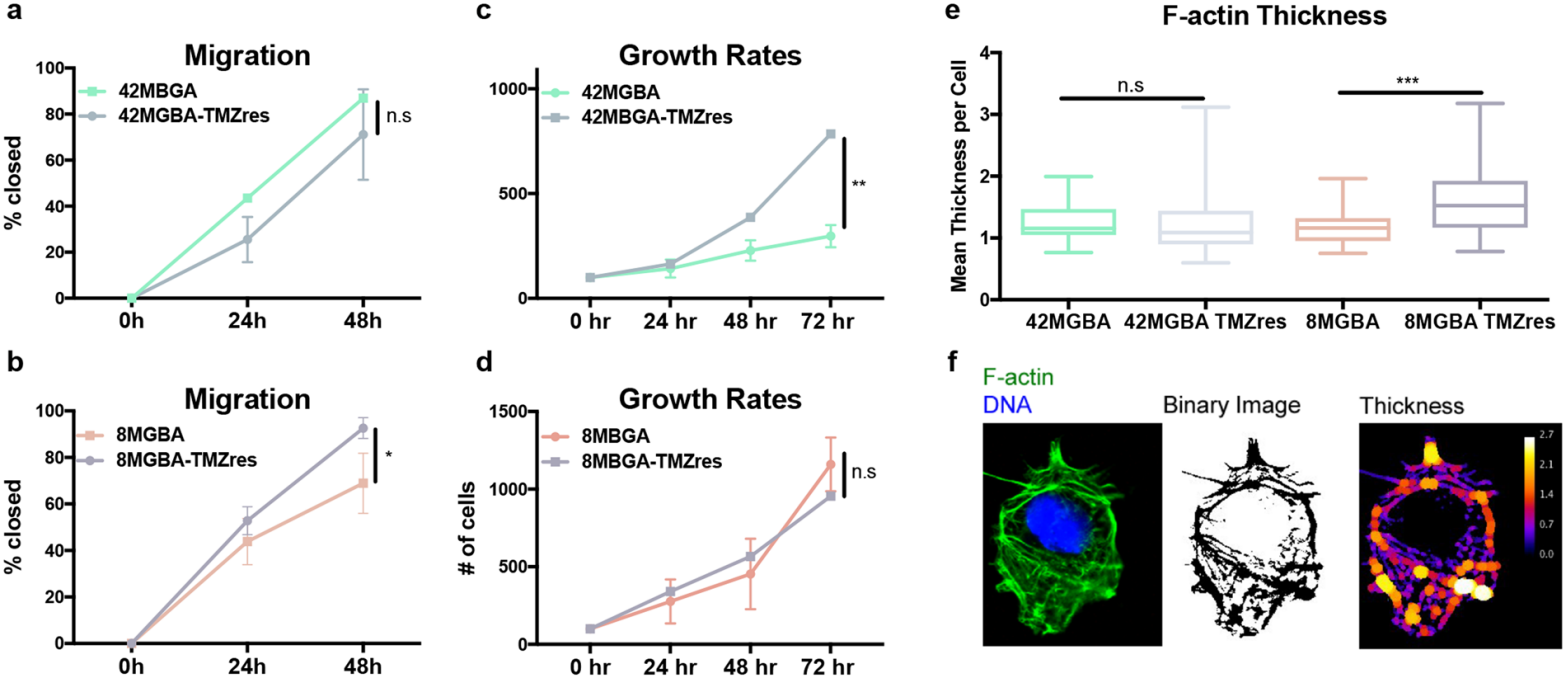
Changes in cell growth, migration, and the actin cytoskeleton. (**a**,**b**) Scratch-wound analysis for 2D migration over 48 hours. t-test at 48 hours 8MBGA p = 0.04. (**c**,**d**) Trypan blue dye exclusion assay to measure cell growth over 72 hours; 42MBGA p = 0.0066. (**e**) Mean thickness of F-actin filaments assessed by FIJI plug-ins as denoted in Methods section. Mann-Whitney U test 8MBGA p = 0.001. (**f**) Representative image of quantification in e.

We then determined how TMZ-resistance affected cell size and proliferative vs migratory phenotypes. 42MBGA-TMZres cell size was not changed vs 42MGBA-WT, though their basal growth rate was dramatically increased (Fig. 4c, Sup Fig. 3a,b). They also showed a modest but nonsignificant reduction in cell migration (Fig. 4a, images in Sup Fig. 4). In contrast, 8MBGA-TMZres cell size was significantly increased when compared to its parental cell line, while the basal growth rate was unchanged (Fig. 4d, Sup Fig. 3a,b). 8MGBA-TMZres cells were significantly more migratory than 8MGBA-WT cells (Fig. 4b). Enhanced cell migration correlated with increased F-actin stress fiber thickness in both TMZres models. There was no significant change in F-actin thickness in the 42MBGA-TMZres compared to 42MGBA-WT cells, while it was significantly increased in the more migratory 8MGBA-TMZres when compared to 8MBGA-WT cells (Fig. 4e,f).

### TMZ resistance leads to an increase in intracellular vesicles and lipid metabolites

**Figure 5:**
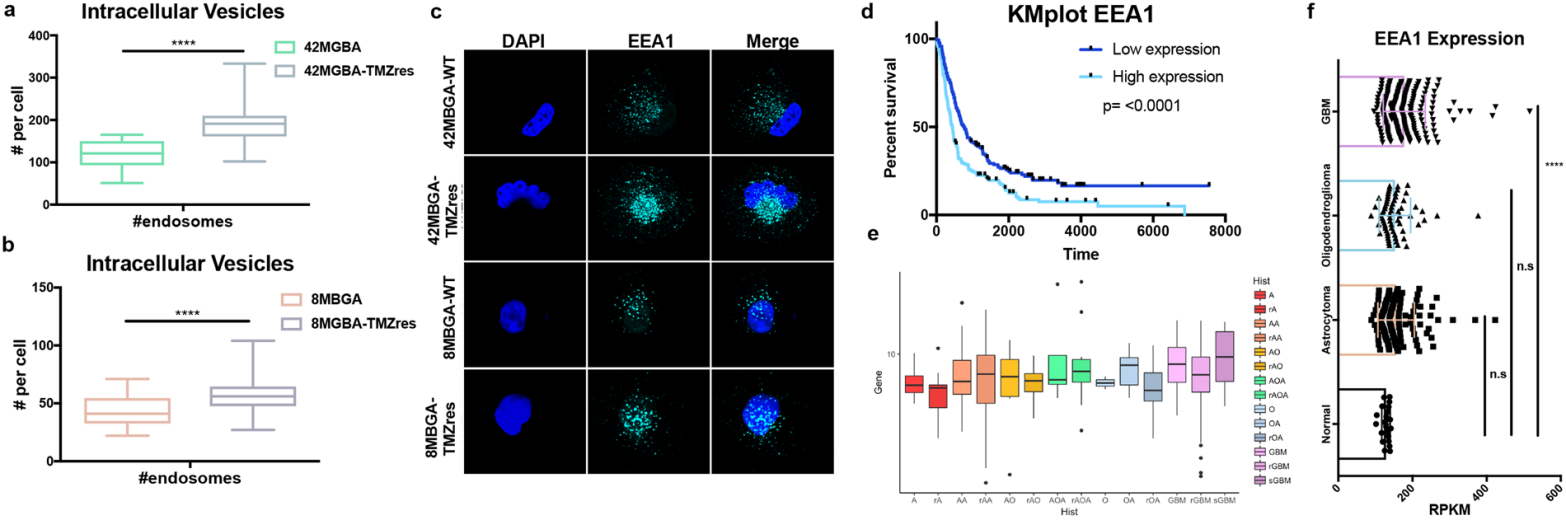
Increase of intracellular vesicles with TMZ resistance. (**a**,**b**) Number of intracellular vesicles stained positive for the early endosomal marker EEA1 quantified between sensitive and resistant cell lines by immunofluorescence. t-test (**a**) 42MGBA p = <0.0001; (**b**) 8MBGA p = <0.0001. (**c**) Representative image of EEA1 immunofluorescence. (**d**) REMBRANDT dataset Kaplan-Meier overall survival plot with median expression of EEA1 in all brain cancer patients. (**e**) EEA1 expression in the Chinese Cancer Genome Consortium (CCGC) dataset by brain cancer type. A = astrocytoma; AA = anaplastic astrocytoma; AO = anaplastic oligodendroglioma; AOA = anaplastic oligoastrocytoma; O = oligodendroglioma; OA = oligoastrocytoma; GBM = glioblastoma. r(cancer type) = recurrent, s(cancer type) = secondary. (**f**) EEA1 expression in the REMBRANDT dataset by cancer type. One-way ANOVA p = <0.0001.

**Figure 6:**
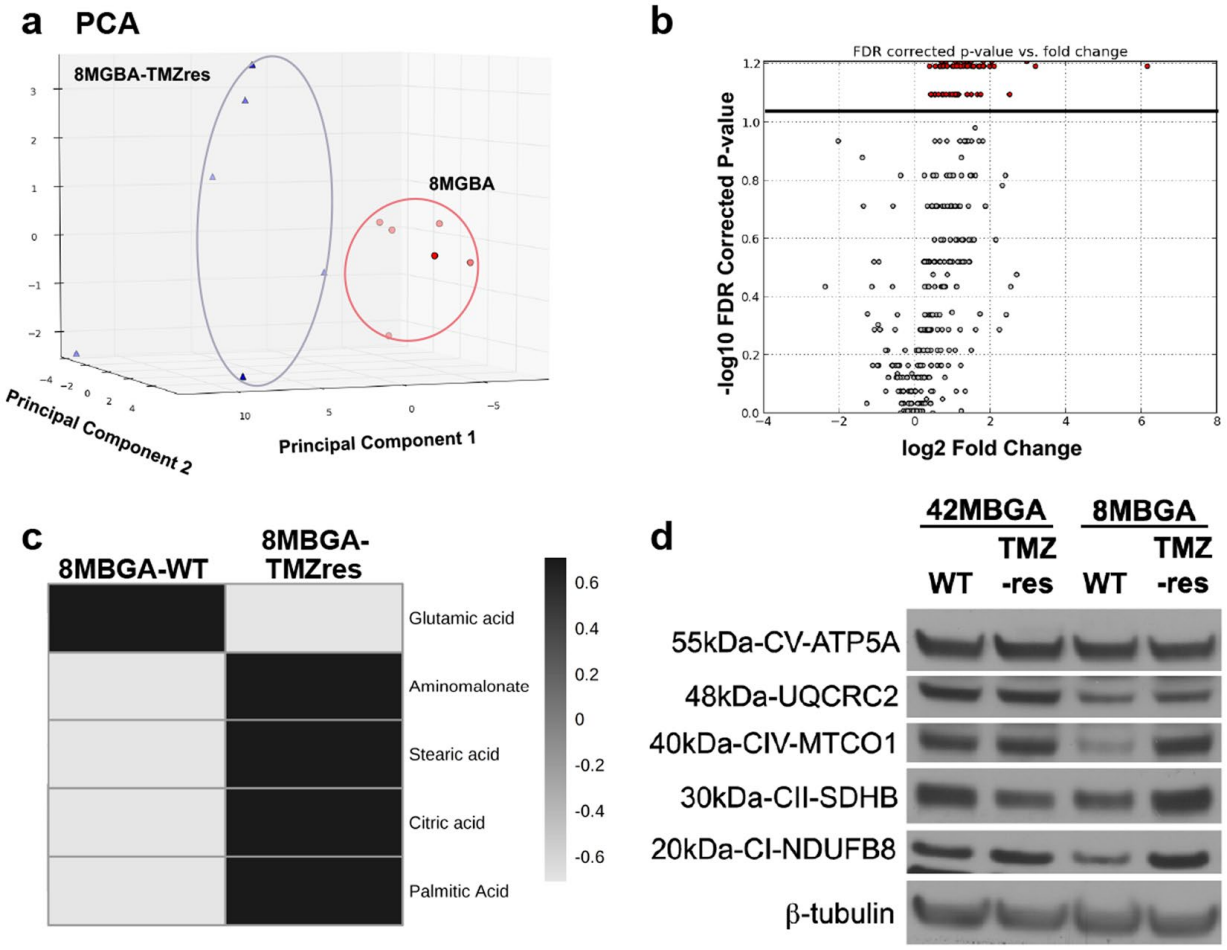
GC-TOF-MS analysis of 8MBGA-WT and –TMZres media. (**a**) PCA plot of metabolites from 8MGBA and 8MGBA-TMZres cell culture media detected by GC-TOF-MS and (**b**) the corresponding volcano plot (significant ions above black line). Heatmap in (**c**) shows significant metabolites between the groups (p = 0.005) and was validated through the retention index value and comparison of the electron impact spectra to the NIST 14 database. Compound averages are shown. (**d**) Altered expression of components of electron transport chain complexes I, II, and IV in 8MGBA-TMZres cells.

An advantage of selecting these resistant models was the ability to observe changes that occurred during and continued to persist after resistance was acquired. One observation was the number of intracellular puncta and extracellular vesicles that were expelled into the media as the cells became resistant to TMZ (Fig. 5c). As there was no longer a dramatic increase in the SubG1 fraction (Fig. 1e, Sup Fig. 1), or cleaved PARP (Fig. 1h) upon resistance, we were able to rule out general apoptotic bodies. We therefore wanted to test if there was an increase in overall vesicle production in the TMZres vs WT cell lines. To accomplish this, we stained for the endosomal marker early endosome antigen 1 (EEA1). After analysis, we saw that there was a significant increase in the total number of endosomes in both TMZres cell lines compared to their parental lines (Fig. 5a–c). Interestingly in two independent datasets – the Chinese Cancer Genomics Consortium (CCGC) and REpository for Molecular BRAin Neoplasia DaTa (REMBRANDT) – EEA1 expression increases from normal brain to GBM, and survival is significantly better for patients with tumors having lower expression of EEA1 (Fig. 5d–f). However, it was not only intracellular, or early endosome, vesicles that we observed to be different, but extracellular vesicles as well. We therefore performed untargeted liquid chromatography and gas chromatography time-of-flight-based mass spectrometry (LC-MS, GC-TOF-MS) on cell culture media from the 8MGBA-WT and 8MBGA-TMZres cell line to identify differences in metabolites that may be associated with this phenotype. Putative results from the LC-MS showed an increase in the extracellular lipids LysoPC(16:0) and LysoPE(16:0) (Sup Fig. 5). Interestingly, indoleacetaldehyde, a product of tryptophan metabolism, was also shown to be putatively increased in the 8MGBA-TMZres media. Tryptophan metabolism disruption has been shown previously as a feature of treatment adaptation in GBM cells^19^. Consistent with our observation of increased extracellular vesicles, there was an increase in palmitic acid and stearic acid lipids in the GC-TOF-MS metabolites from the 8MBGA-TMZres cell line conditioned media (Fig. 6a–c). The top hit from GC-TOF-MS was the non-aminogenic amino acid aminomalonate, an inhibitor of aminolevulinic acid synthase, the enzyme that catalyzes the rate limiting step of the protoporphyrin IX (PpI IX)/heme synthesis pathway^20^. This pathway is deregulated in many cancers, including GBM, and can be exploited to identify tumor area during surgery^21,22^. The last significant metabolite was citric acid, which has been associated with a decrease in overall survival in GBM patients with higher citric acid in their cerebral spinal fluid^23^.

The enrichment of lipid metabolites and aminomalonate identified in conditioned media from resistant cells by LC-MS and GC-TOF-MS, respectively, could suggest dysregulation of mitochondrial metabolism. We therefore determined whether the expression of electron transport chain (ETC) subunits is altered between sensitive and resistant lines. There were no changes between ETC subunits in the 42MBGA-WT and -TMZres lines. However, there was an increase in three ETC subunits in the 8MBGA-TMZres cell line as compared to its WT control: MT-CO1 (Complex IV), SDHB (Complex II), and NDUFB8 (Complex I) (Fig. 6d). Increased expression of Complex IV and Complex II subunits is consistent with prior studies^24^, while in other malignancies, inactivating mutations in SDHB (Complex II) are implicated in TMZ responsiveness^25,26^.

## Discussion

In the present study, we created two new GBM cell lines that are resistant to the standard of care drug TMZ *in vitro*. True to the heterogeneous nature of clinical GBM, the phenotypes displayed by these resistant cells were similar, but not identical. TMZ treatment no longer induced G2/M arrest in either of these cell lines, and both were markedly less responsive to BCNU, the previous standard of care for GBM. Both resistant cell lines also gained expression of the MGMT protein, which is responsible for removing one of the DNA adducts caused by TMZ treatment^5^.

An important finding of the present study is that both TMZres models have severely impaired response to BCNU. For decades BCNU was the standard of care for GBM, and it is still a component of some second-line regimens. Findings from clinical studies examining the efficacy of BCNU in recurrent GBM post-TMZ treatment are inconsistent, with some suggesting BCNU remains active while others demonstrate no benefit^16–18^. These TMZres cell line pairs may therefore also prove to be valuable resources for defining contexts in which BCNU is or is not appropriate second-line treatment, and for identifying other, more appropriate therapies for TMZ-resistant GBM.

A key advantage provided by creating these resistant cell lines *in vitro* as opposed to isolation from a resistant tumor is the ability to directly compare them to their respective sensitive parental lines. There was an increase in nuclear size in both resistant cell lines when compared with their parental cell lines. In the 42MGBA-TMZres cell line there was also a change in nuclear morphology which has been described in other GBM cell lines that have acquired resistance to TMZ^27,28^. This may be associated with stability of the resistant phenotype, as we observed that the 42MBGA-TMZres cell line resistant phenotype can be maintained without continuous TMZ treatment. However, the 8MGBA-TMZres cell line, which has not undergone nuclear structure reorganization, and showed two distinct populations with respect to metaphase spreads, regained partial sensitivity when cultured in the absence of TMZ.

Increases in nuclear size can be driven by increased chromosome numbers or impaired transport of mRNA or proteins out of the nucleus^29^. In these novel TMZ resistant models, we hypothesized that the increase in nuclear size was a function of retained chromosomes. This is consistent with the mechanism of action of TMZ, which induces a G2/M arrest in cells. Cells which escape TMZ-mediated cell cycle arrest and death have already replicated their DNA, and may therefore retain sister chromatids that were not able to separate during anaphase. Because both parental GBM cell lines were initially polyploid with distinct karyotypes, we assessed two different chromosomes – 17 for the 8MBGA-WT and –TMZres cell lines, and X for the 42MBGA-WT and –TMZres cell lines – as an indicator for chromosomal copy number alterations. Both TMZ resistant variants showed significant copy number gains. Of note, our chromosome 17 locus specific probe targets the *STAT5* gene, which has already been implicated as a pro-tumorigenic factor in GBM^30^. The increased copy number of the *STAT5* locus in the 8MBGA-TMZres cell line is consistent with previous work and suggests that the observed increase in nuclear size is at least partially attributable to an increase in chromosome number. Because chromosome 17 existed in multiple copies in the 42MBGA-WT cell line, we chose an X-centromeric probe to test chromosomal copy number changes in the 42MBGA-TMZres line. Again, we saw an increase in X-chromosome copy number in the 42MBGA-TMZres line, and an overall increase in the total number of chromosomes. While it can be energetically disadvantageous to increase ploidy, we hypothesize that, as for STAT5, there may be X chromosome genes that when duplicated give a proliferative advantage to these cells.

Two major challenges in the clinical management of GBM, particularly after TMZ resistance has developed, are rapid tumor cell proliferation and widespread local migration or invasion^31^. However, these phenotypes are rarely displayed by the same cells, giving rise to the “go or grow” hypothesis to explain aggressive behavior and drug resistance^28^. Our novel *in vitro* models of TMZ-resistant GBM provide examples of both phenotypes. The 42MBGA-TMZres cell line did not exhibit any significant changes in migration, cytoskeletal structure, or cell size when compared to its parental cell line, but it did proliferate significantly faster. In contrast, the 8MBGA-TMZres cell line had a significantly increased migratory capacity, F-actin thickness, and overall cell size relative to 8MGBA-WT cells, but no accompanying increase in proliferation. These divergent alterations in basal growth rate and migratory capacity provide two unique models – one adapting the “go” (8MGBA-TMZres) and the other the “grow” (42MBGA-TMZres) strategy of TMZ resistance^13^. There have been many hypotheses as to what determines the switch between “go or grow”, including mutational status, hypoxia, and influences of the surrounding cell secretome^13,32,33^. Activation of the FAK and MAPK/ERK pathways have been shown to be differentially regulated between a “go” and “grow” phenotype^32,33^, respectively. Others have also hypothesized that remaining cells from the “go” population lead to recurrence of GBM^13^. Therefore, more insight needs to be gained into potential common pathways between the “go or grow” phenotypes that could be used as therapeutic targets for both cell populations. Candidate strategies to revert “go” phenotypes could include inhibition of the integrin signaling axis, either through blockade of integrin engagement^34,35^ or inhibition of FAK^36^, while MEK inhibition could suppress the “grow” phenotype^37,38^. These four cell lines are therefore valuable tools that can be used to provide important insight into the differential mechanisms of TMZ resistance.

Acquisition of TMZ resistance was associated with a marked increase in the number of intracellular and extracellular vesicles in our models. While there are always potential limitations to studying metabolism in an *in vitro* setting, Oliveira *et al*. showed that the *in vitro* secretome can affect the “go or grow” phenotype of murine GBM cells^32^. Therefore is it important to identify potential *in vitro* mechanisms that can be further extrapolated to *in vivo* models in the future. The increase in early endosome marker EEA1 expression is interesting, as others have shown EEA1 to interact directly with RRAD, STAT3, and EGFR to regulate EGFR’s subcellular localization and resistance to TMZ^39^. Clinically, higher EEA1 mRNA expression is associated with poor survival. EGFR and its structural variants have been targeted extensively in GBM, but with limited success. We suggest further study of the mechanisms by which EEA1 contributes to the TMZ resistant phenotype could provide a new way to target EGFR by modifying its subcellular localization rather than activation^40^. In addition to an accumulation of early endosomes, we observed vesicles expelled into the media of the resistant cell lines. Others have shown that TMZ-resistant glioma stem cells release more exosomes than their sensitive counterparts, which was consistent with our *in vitro* created models^41^. This was reflected in the putative increase of lipids from the LC-MS analysis. Another hit, indoleacetaldehyde, is a product of tryptophan metabolism, hyperactivation of which occurs in GBM and is thought to suppress anti-tumor immunity^19^. Extracellular lipids, stearic and palmitic acid, were also found and validated in the GC-TOF-MS analysis along with citric acid. Citric acid is a key regulator of metabolism, and when increased in the cerebral spinal fluid of GBM patients confers a lower overall survival^23^. The top hit from GC-TOF-MS was the non-aminogenic amino acid aminomalonate. Aminomalonate inhibits aminolevulinic acid synthase, the rate-limiting enzyme of the protophorphyrin IX (PpI IX)/heme synthesis pathway^20^. PpI IX pathway activity is exploited for intra-operative imaging in GBM, and has more recently been used for potential therapeutic gain^42,43^. Defining the drivers of aminomalonate secretion in our TMZ-resistant models may be an important future strategy to identify when PpI IX pathway-based imaging and therapeutic approaches may be warranted.

**Figure 7:**
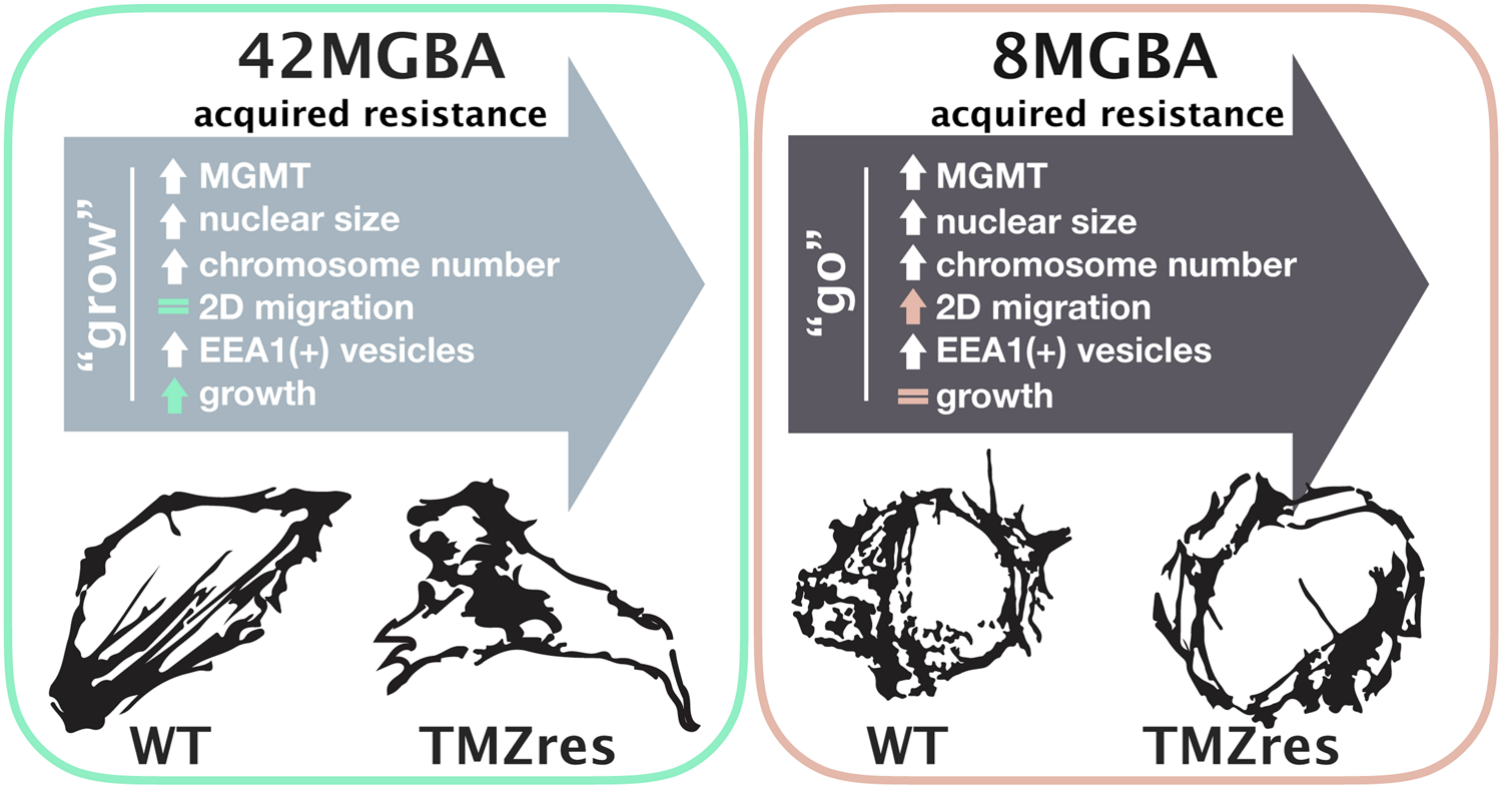
Summary figure of changes between wild-type (WT) cell lines upon acquired TMZ resistance (TMZres).

In conclusion, we present two novel TMZ-resistant GBM cell lines from both a male and female patient that have distinct proliferative and migratory capacities while sharing certain GBM resistance characteristics such as increased chromosome number, nuclear size, and vesicle formation (Fig. 7). These models will be useful tools for the field to better define essential drug resistance mechanisms and therapies in TMZ-resistant GBM.

## Methods

### Cell Lines and Culturing Conditions

42MBGA and 8MGBA cell lines were provided by Dr. Jeffrey Toretsky (Lombardi Comprehensive Cancer Center (LCCC), Georgetown University, Washington DC). 42MBGA-TMZres and 8MGBA-TMZres were developed by our lab with constant exposure of the parental cell lines to increasing concentrations of TMZ, from 12.5, 25, 50, 100, 200 µM, over the course of 2–3 months. All cells tested negative for *Mycoplasma* contamination, and were maintained in a humidified incubator with 95% air: 5% carbon dioxide. All cell lines were fingerprinted by the LCCC Tissue Culture Shared Resource to verify their authenticity using the standard 9 STR loci and Y-specific amelogenin. Both the 42MBGA-TMZres and 8MGBA-TMZres fingerprinted the same as their parental cell line. 42MGBA and 8MBGA cells were grown in DMEM with 10% FBS. 42MBGA-TMZres and 8MBGA-TMZres cells were grown in DMEM with 10% FBS and 100 µM TMZ. TMZ (Selleckchem, Catalog No. S1237) was dissolved in DMSO to 130 mM and used at concentrations indicated. Bis-chloroethylnitrosourea (BCNU) was a kind gift from Dr. Esther Chang, and was dissolved in ethanol to 100 mM before use at concentrations indicated.

### Cell Cycle Analysis

On day 0, cells were seeded at 100,000–150,000 cells per well in 6-well plastic tissue culture dishes one day prior to treatment with the indicated concentrations of drug. For experiments with TMZ or BCNU cells were treated for 72 hours. After 72 hour treatments, cells were collected, ethanol-fixed, stained with propidium iodide, and analyzed for cell subG1 (fragmented) DNA content and cell cycle profile by fluorescence activated cell sorting.

### Western Blotting

Cells were lysed in RIPA buffer for protein extractions and separated by a 4–12% gradient gel (Novex by Life Tech, NP0321BOX). They were then transferred onto Nitrocellulose membranes (Invitrogen, IB23001) with the iBlot 2 and probed with antibodies against MGMT (1:1000, Cell Signaling, 2739S), Beta-Tubulin (1:10,000,Sigma Aldrich, T7816), PARP (1:1000, Cell Signaling, 9542L), Actin (1:5000, Sigma Aldrich, A5316), and Electron Transport Chain antibody cocktail (1:500, Abcam, ab110413). Proteins were detected with horseradish peroxidase-conjugated secondary antibodies (1:5000, GE Healthcare Life Sciences) and enhanced chemiluminescent detection HyGLO Quick Spray Chemiluminescent (Denville) for dark room development.

### Evaluation of chromosomal copy number changes induced by TMZ treatment

Metaphase and interphase slide preparation was done using a standard protocol^44^. For metaphase chromosomal copy number determination, chromosomes were stained with 4′,6-diamidino-2-phenylindole (DAPI). FISH analysis was performed using a standard protocol^45–47^. A 17q21.1-21.2 locus-specific FISH probe (Stat5A/B locus) was used to evaluate chromosome 17 copy number change in the 8MGBA-TMZres and WT cell lines, as described earlier^45^. For the X chromosome enumeration in the 42MGBA-TMZres and WT cell lines, an X chromosome centromeric probe obtained from Empire Genomics (Buffalo, NY) was used according a manufacturer’s protocol. Scoring of cells and digital image acquisition were performed using a Leica DMRBE microscope (Leica Microsystems, GmbH, Wetzlar, Germany) equipped with the appropriate optical filters and a CCD camera^45^.

### Scratch Wound Assays

Cells were plated at 150,000–200,000 cells/well and allowed 48 hours to create a monolayer. After monolayer formation, a P200 tip was used to make the scratch. Images were taken at 0 hr, 24 hr, and 48 hr time points. Analysis was done in ImageJ^48^ to determine percent closed with 0% being at 0 hr.

### F-actin measurements

FIJI (ImageJ^48^) was used to measure the thickness of F-actin fibers. Grayscale images of phalloidin-stained cells were converted to binary using the Threshold tool (black/white). The BoneJ plugin^49^ was then used to measure fiber thickness in pixels. The mean thickness per cell is reported for 28-29 individual cells per cell line.

### Immunofluorescent Staining

8MGBA-WT and 8MGBA-TMZres cells were seeded at a density of 50,000 cells onto 18 mm coverslips in 12-well dishes. On the following day, the media was removed and cells were fixed and permeabilized in 3.2% paraformaldehyde (PFA) with 0.2% Triton X-100 in PBS for 5 minutes at room temperature. Three washes were performed with PBS in the 12-well plate, then coverslips were inverted onto 100 μl of primary antibody in the antibody block (0.1% gelatin with 10% normal donkey serum in PBS) on strips of parafilm and incubated for one hour. Coverslips were first stained with EEA1 (Cell Signaling, 3288), an early endosomal marker (1:100 dilution, 1 hr). Phalloidin (polymerized F-actin, Invitrogen, A12379, 1:200 dilution), and DAPI (DNA, 1:500 dilution) stains were added to the secondary antibody mixtures. Each coverslip was washed three times with PBS after primary incubation and then were inverted onto 100 µL of the appropriate secondary antibody, DAPI dihydrochloride, and (where appropriate) ActiStain-488-phalloidin (Cytoskeleton, Denver, CO) in antibody block in the dark for 20 minutes. Coverslips were again washed 3x with PBS, then gently dipped three times into molecular biology-grade water before inversion onto one drop of FLUOROGEL (Electron Microscopy Sciences, Hatfield, PA) then allowed to air-dry in the dark for at least 10 minutes. Slides were stored at 4 °C until image collection on the LCCC Microscopy & Imaging Shared Resource’s Leica SP8 microscope with the 63x oil objective.

### Metabolomics

#### Ultra-performance liquid chromatography (UPLC) quadrupole time-of-flight (QTOF) mass spectrometry (MS)

Cell culture media (500 µL) was concentrated to 25 µL under N_2_. The 25 µL sample was deproteinized using cold acetonitrile (40%): methanol (25%): water (35%) (175 µL) containing internal standards (2 µM debrisoquine sulfate, 30 µM 4-nitrobenzoic acid). All solvents used were LC-MS grade (Fisher Scientific, Hanover Park, IL). Samples were vortexed and incubated on ice for 10 min. Incubated samples were centrifuged at 13000 rpm for 20 min at 4 °C. The supernatant was evaporated in a speed-vac with no heat. Dried samples were kept at −80 °C until analysis. For analysis, the dried samples were reconstituted in acetonitrile (1%): methanol (5%): water (94%) (200 µL). Samples were centrifuged for 20 min at 4 °C and the supernatant transferred to a vial for LC-MS analysis. Briefly, samples were injected (2 μL) into a Waters Acquity UPLC system coupled to a Xevo^®^ G2 QTOF-MS using Leucine enkephalin as Lockspray^®^ for accurate mass calibration. Data was processed, aligned, and analyzed using MassLynx^TM^ (Waters, Milford, MA), XCMS online^50^, Metaboanalyst, and R^51^.

#### Gas chromatography time-of-flight mass spectrometry (GC-TOF-MS)

Internal standards (1 µM 4-nitrobenzoic acid) were added to culture media (500 μL) that was deproteinized as above and then evaporated in a speed-vac and transferred to a GC vial with a silanized insert. Derivatization and injection was performed with a Gerstel (Linthicum, MD) autosampler with automated liner exchange and cooled injection system. The dry residue was derivatized with 20 μL O-Methoxyamine-Hydrochloride (MOX; Thermo Scientific prod # TS-45950) at 40 °C with constant agitation (30 min), and then with 80 μL of N-Methyl-N-(trimethylsilyl) trifluoroacetamide (MSTFA)/with 1% trimethychlorosilane (TMCS) (Thermo Scientific prod # TS48915) at 40 °C with constant agitation (30 min). Samples were incubated at 20 °C for 4 hrs before injection (1.5 μL) for GC-TOF-MS analysis.

Samples were injected in split mode (1:5) into an Agilent 7890B GC system (Santa Clara, CA, USA) that was coupled to a Pegasus HT TOF-MS (LECO Corporation, St. Joseph, MI, USA). Separation was achieved on a Rtx-5 w/Integra-Guard capillary column (5% diphenyl/95% dimethyl polysiloxane; 30 m × 0.25 mm ID, 0.25 μm film thickness; Restek Corporation, Bellefonte, PA, USA), with helium as the carrier gas at a constant flow rate of 1.0 mL/min. The temperature of the inlet, transfer line, and ion source was set to 150, 270, and 230 °C, respectively. The GC temperature programming was set to 0.2 min of isothermal heating at 70 °C, followed by 10 °C/min ramp to 270 °C, a 4.0 min isothermal heating of 270 °C, 20 °C/min ramp to 320 °C, and a 2.0 min isothermal heating of 320 °C as previously described^52^. Electron impact ionization (70 eV) at full scan mode (*m/z* 40–600) was used, with an acquisition rate of 30 spectra/sec. Fatty acid methyl esters (C_4_–C_24_ FAMEs; Sigma-Aldrich), alkanes (alkane standard mix [C_10_–C_40_]; Sigma-Aldrich), and quality control standards (oxalic acid, malonic acid, malic acid, citric acid, methionine, and 2-butenedioic acid) were run to ensure reproducibility of retention indices and derivatization. Data was processed, aligned, and analyzed using ChromaTOF v. 4.51.6.0 with the statistical compare function (Leco, St. Joseph, MI), Metaboanalyst, and in house software MetaboLyzer^53^ as previously described^52^.

### Crystal violet assay

Cells were seeded at a density of 1,000 cells per well in 3, 96-well plastic tissue culture dishes per cell line on day 0. On day 1, one plate was stained with crystal violet (Sigma,C0775). For staining, plates were rinsed 1 time with 1X PBS to remove excess cellular debris. After, 100 µL of 3.2% PFA was added to each well and incubated at room temperature for 5 minutes followed by three washes in 1X PBS. Then, 200 µL of 0.5% crystal violet in 25% methanol was added to each well and incubated at 4 C for 10 min. The stain was then removed and the plate was rinsed 4–6 × with diH2O to remove excess stain. The plates were left to air-dry overnight. On day 8 and 10, all plates were rehydrated with 100% methanol and read at an absorbance of 550 nm.

### Cell size quantification and growth

Cell size was determined by the Countess 2 (Invitrogen) using trypan blue dye exclusion of dead cells. The Countess 2 was also used to determine number of cells for 72-hour growth assays. 100,000 cells were seeded per 6-well on day 0. 24, 48, and 72 hours post-seeding, cells were collected and total cell number was determined by trypan blue dye exclusion.

### Statistical analysis

Results are represented as mean values ± standard error (SD) and considered statistically significant where p-value < 0.05. Three independent biological replicates were done for each experiment, with the exception of metabolomics where six independent replicates were performed. Statistical significance has been calculated using GraphPad Prism version 7.00 for Mac, GraphPad Software, La Jolla California USA (www.graphpad.com) for the Student’s t-test, Mann Whitney U test, one-way ANOVA with Dunnett’s correction, or χ2 analysis of 2 × 3 contingency table. The RStudio packages survival, GGally, ggplot2, readr, and magrittr were used for violin plots and big data sets (CCGA, REMBRANDT)^51^.

## Electronic supplementary material

Supplementary Figures
