# Supplementary material for: Alterations in Cell Motility, Proliferation, and Metabolism in Novel Models of Acquired Temozolomide Resistant Glioblastoma
Source: Sci Rep. 2018 May 8;8:7222. doi: 10.1038/s41598-018-25588-1 (PMC5940876; doi:10.1038/s41598-018-25588-1)

Alterations in Cell Motility, Proliferation, and Metabolism in Novel Models of Acquired Temozolomide Resistant Glioblastoma

Tiek DM[1,2], Rone JD[1], Graham GT[1], Pannkuk EL[1], Haddad BR[1], Riggins RB[1,2]

[1]Department of Oncology, Lombardi Comprehensive Cancer Center, Georgetown University Medical Center, Washington, DC, USA

[2]Correspondence should be addressed to DM Tiek (dmt53@georgetown.edu) and RB Riggins (rbr7@georgetown.edu)

Address: Georgetown University, 3970 Reservoir Road NW, E412 Research Bldg., Washington, DC 20057

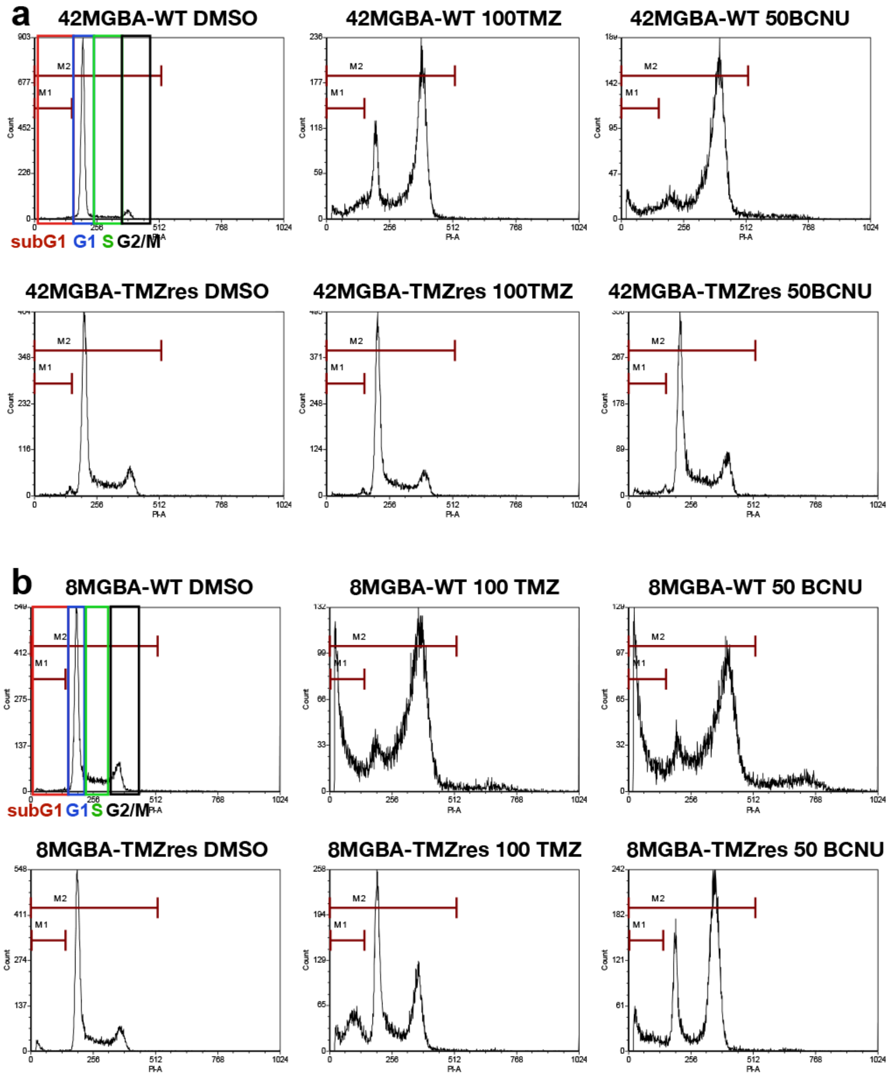

Supplementary Figure 1. Gating and SubG1 peak for cell cycle analysis for a) 42MBGA-WT and –TMZres; b) 8MBGA-WT and –TMZres. M1 is the cut-off for SubG1 fragmented DNA; red box. M2 shows gating of live cells, with the first peak being the G1 fraction; blue box. The second peak represents the G2 fraction; black box, with the space in the middle showing S phase content; green box.

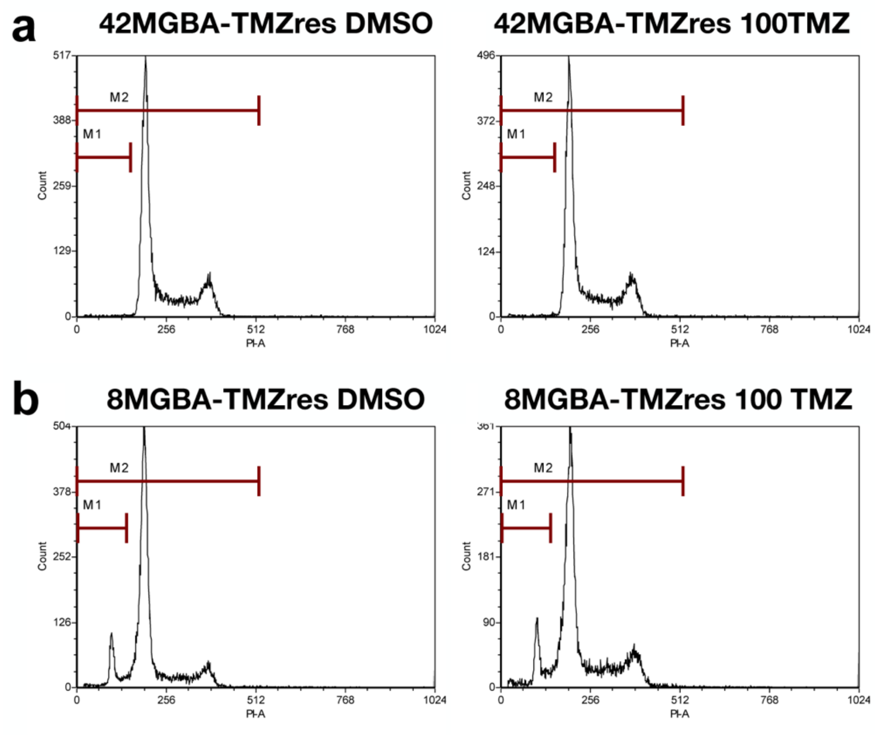

Supplementary Figure 2. TMZ re-challenge. TMZ was removed from the media for three weeks. Cells were then re-challenged with 100 uM TMZ for 72 hours. a) 42MBGA-TMZres had no change in cell cycle +/- TMZ. b) 8MBGA-TMZres cell line had a slight increase in G2/M phase when re-challenged with TMZ, and higher basal levels of SubG1. Same gating as Sup Figure 1. c,d) Quantification of a,b.

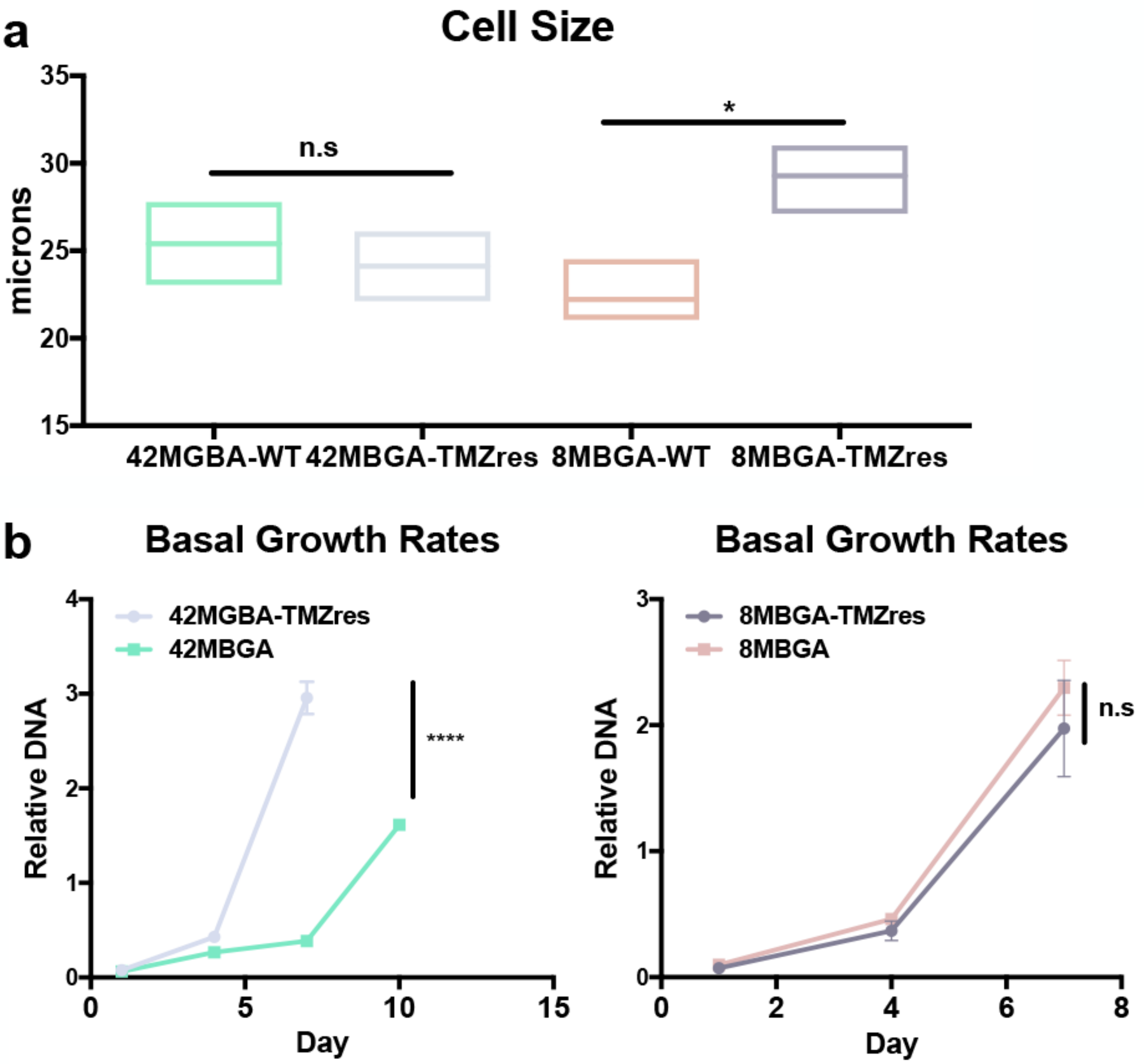

Supplementary Figure 3. Cell size and proliferation rates. a) Cell size in microns by the Countess 2. t-test 8MBGA p = 0.0121 b) Crystal Violet assay of basal proliferation. Area under the curve 42MBGA-TMZres p = <0.0001.

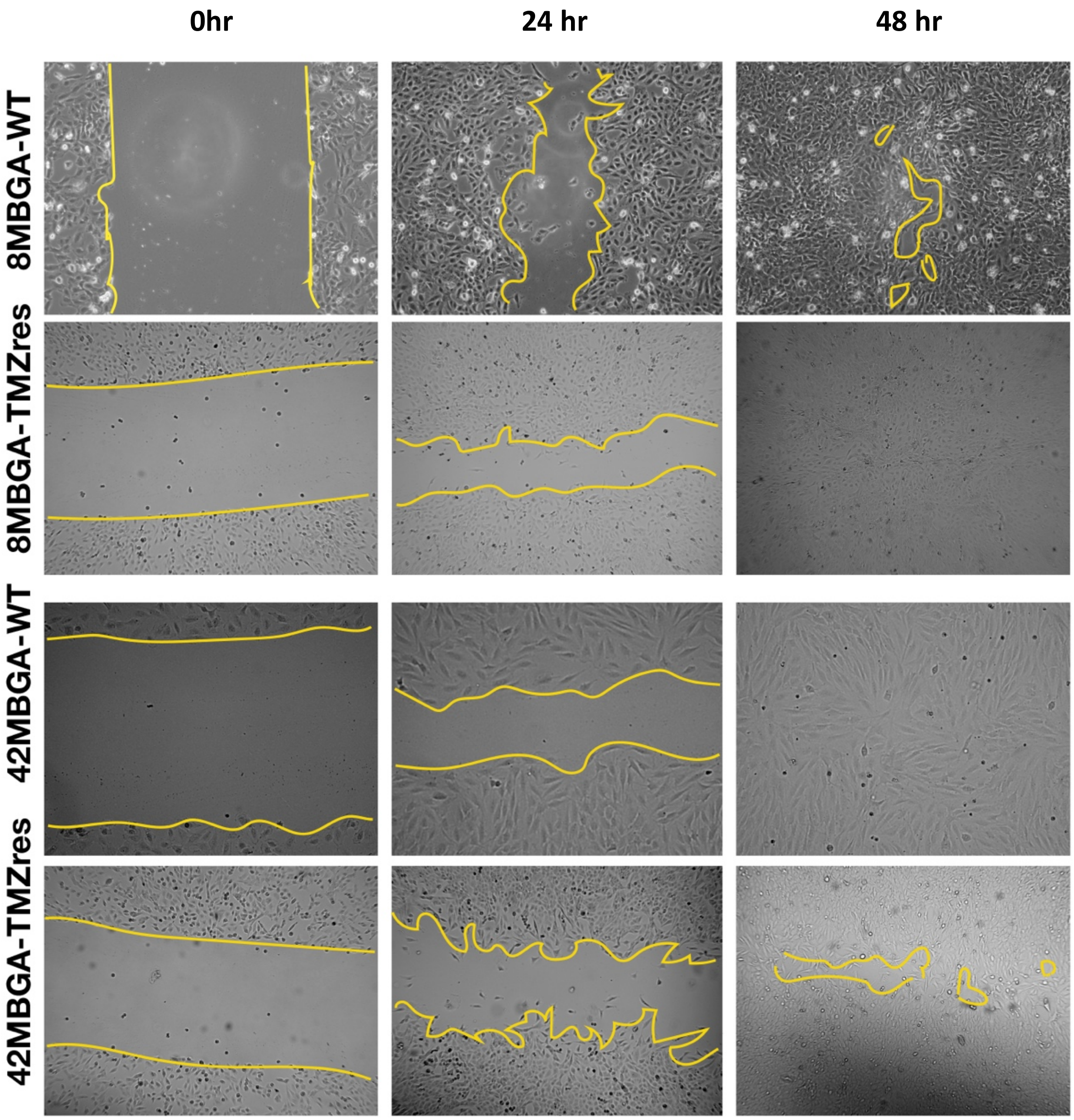

Supplementary Figure 4. Representative images from scratch wound assays. Cell line depicted on the left with time after scratch on the top. Yellow lines denote the edges of open area.

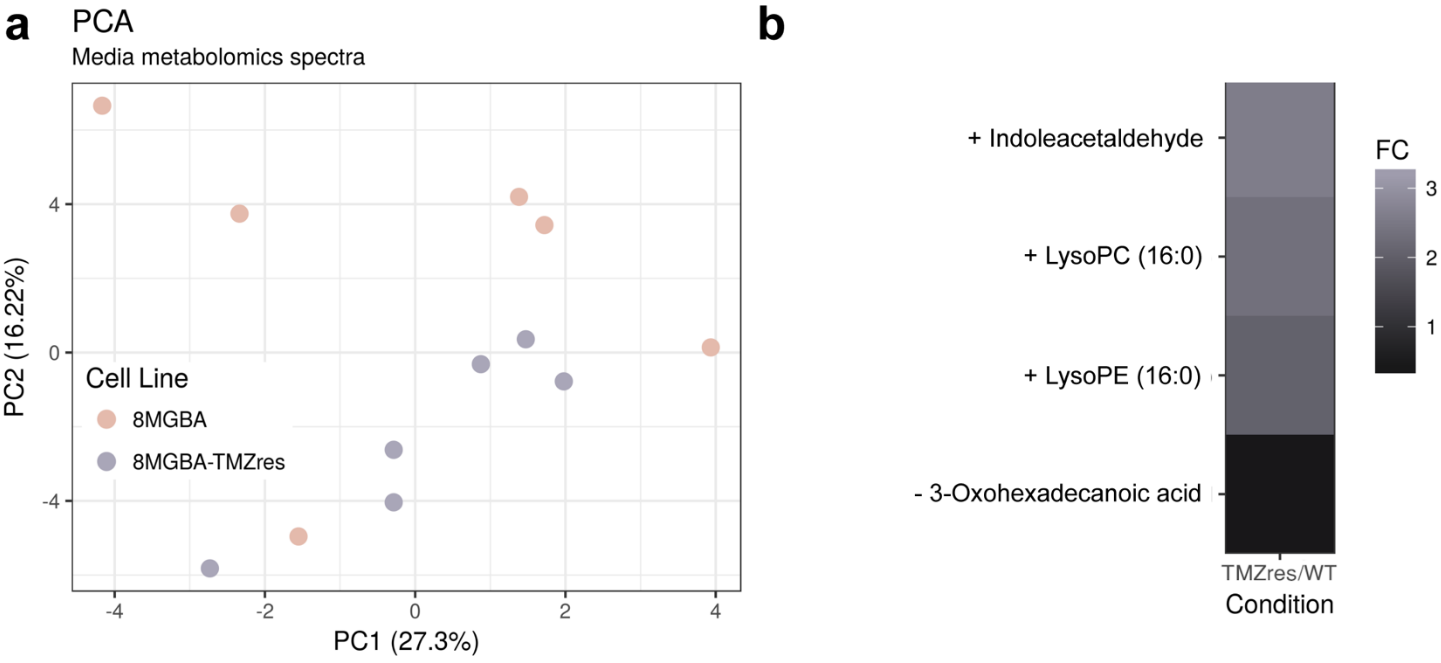

Supplementary Figure 5. Putative LC-MS metabolomics analysis of cell media. a) PCA analysis of LC-MS cell media from 8MBGA-WT vs. –TMZres. b) Heatmap of fold change between TMZres and WT 8MBGA metabolites (+) denotes positive mode, (-) denotes negative mode. Indoleacetaldeyde (p = 0.014); LysoPC (16:0) (p = 0.142); LysoPE (16:0) (p = 0.002); 3-Oxohexadecanoic acid (p = 0.001)).

Figure 1f full blots. Lanes are 42MBGA-WT; 42MBGA-TMZres; 8MBGA-WT; 8MBGA-TMZres; T98G. T98G's were cropped off, as they are not a part of this paper and were a positive control for the MGMT protein.

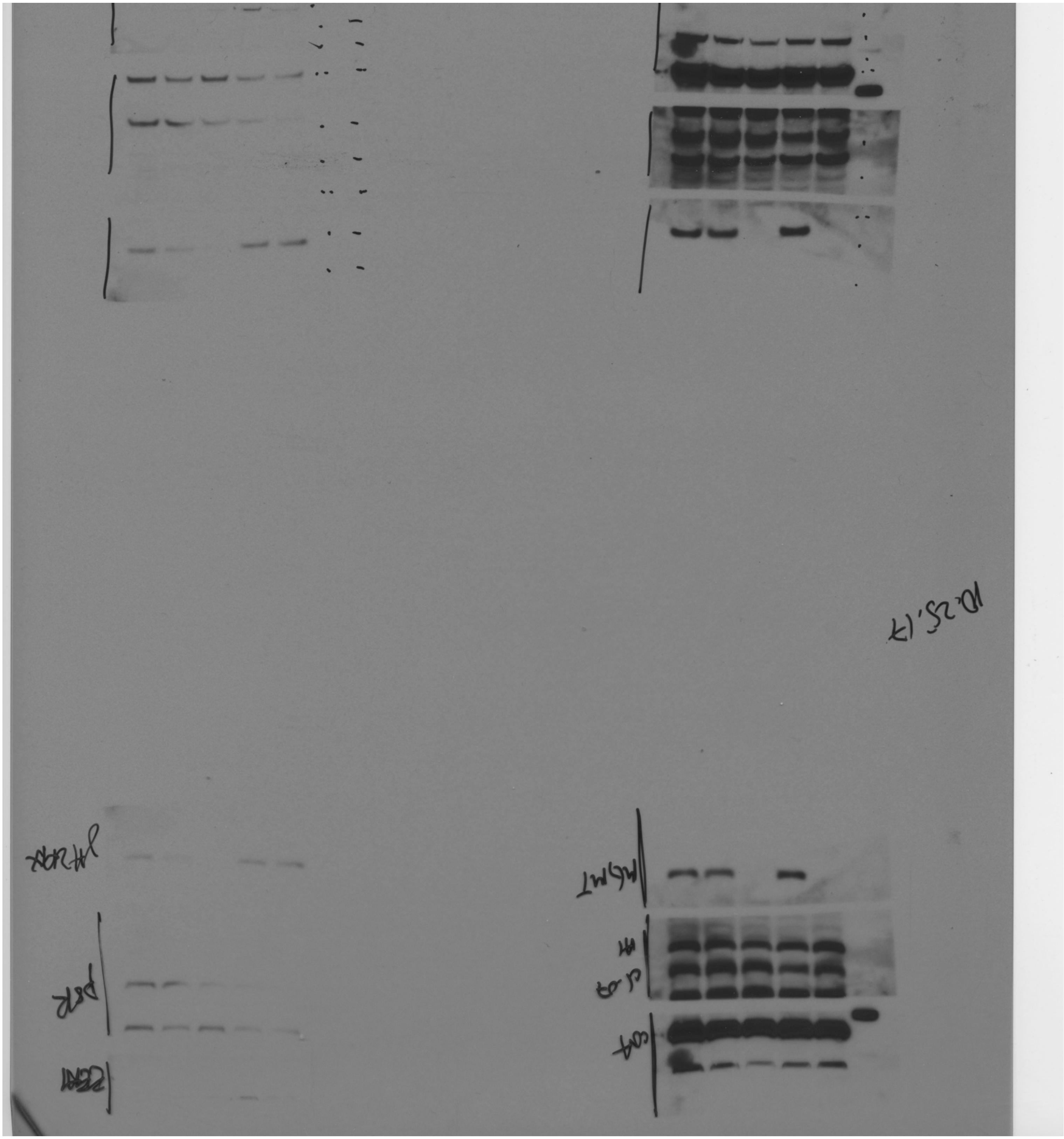

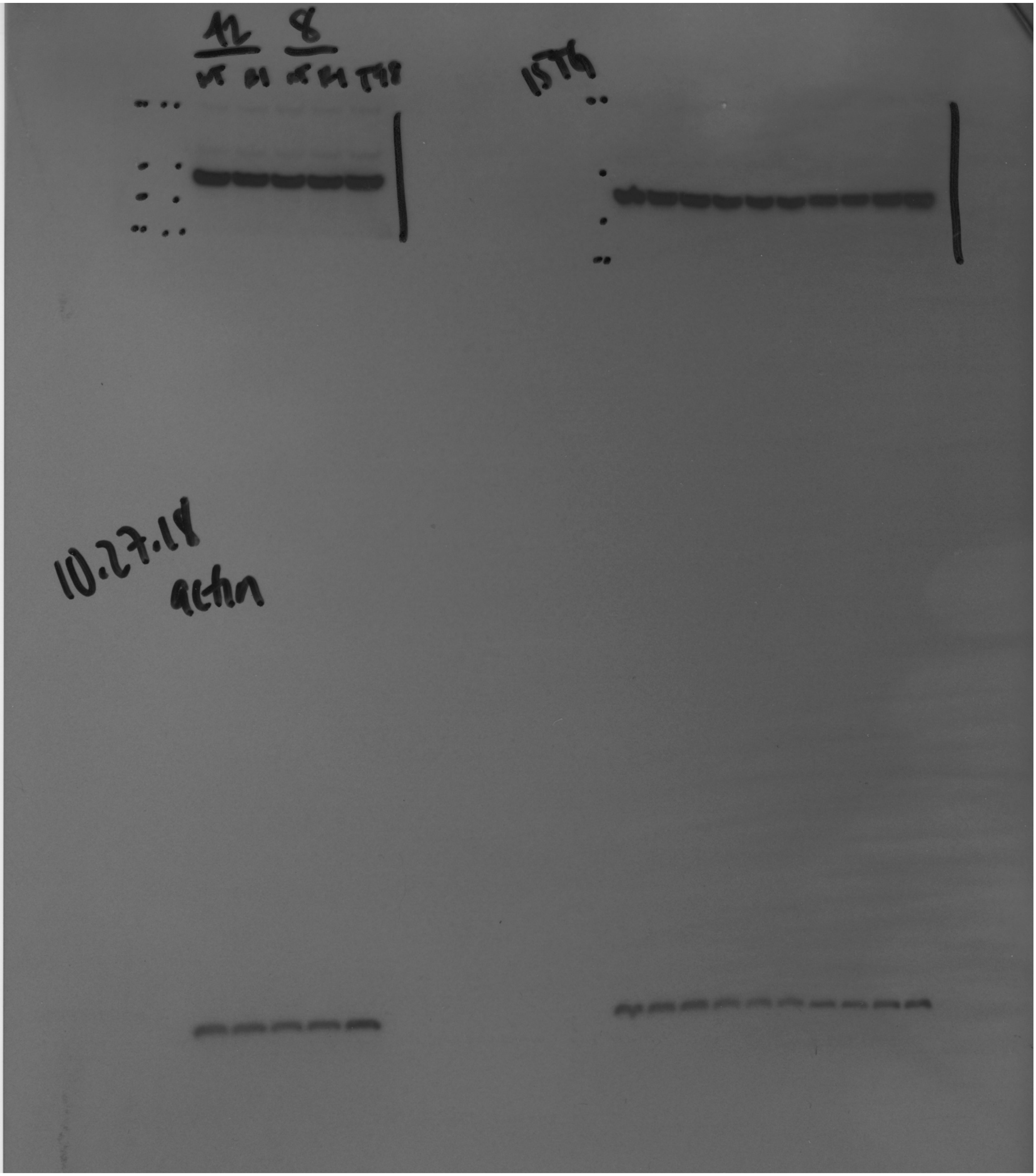
12
8
10.27.18
actin

Figure 1h full blots.

PARP (full and cleaved) was taken at two separate exposure as loading was normalized between cell lines, not across cell lines. Samples were run on two different gels splitting the lysate evenly. Dox lanes were cropped out as they were not relevant to this publication. Lanes are: 8MBGA-WT(DMSO, TMZ, BCNU, Doxorubicin) 8MBGA-TMZres(DMSO, TMZ, BCNU, Doxorubicin).

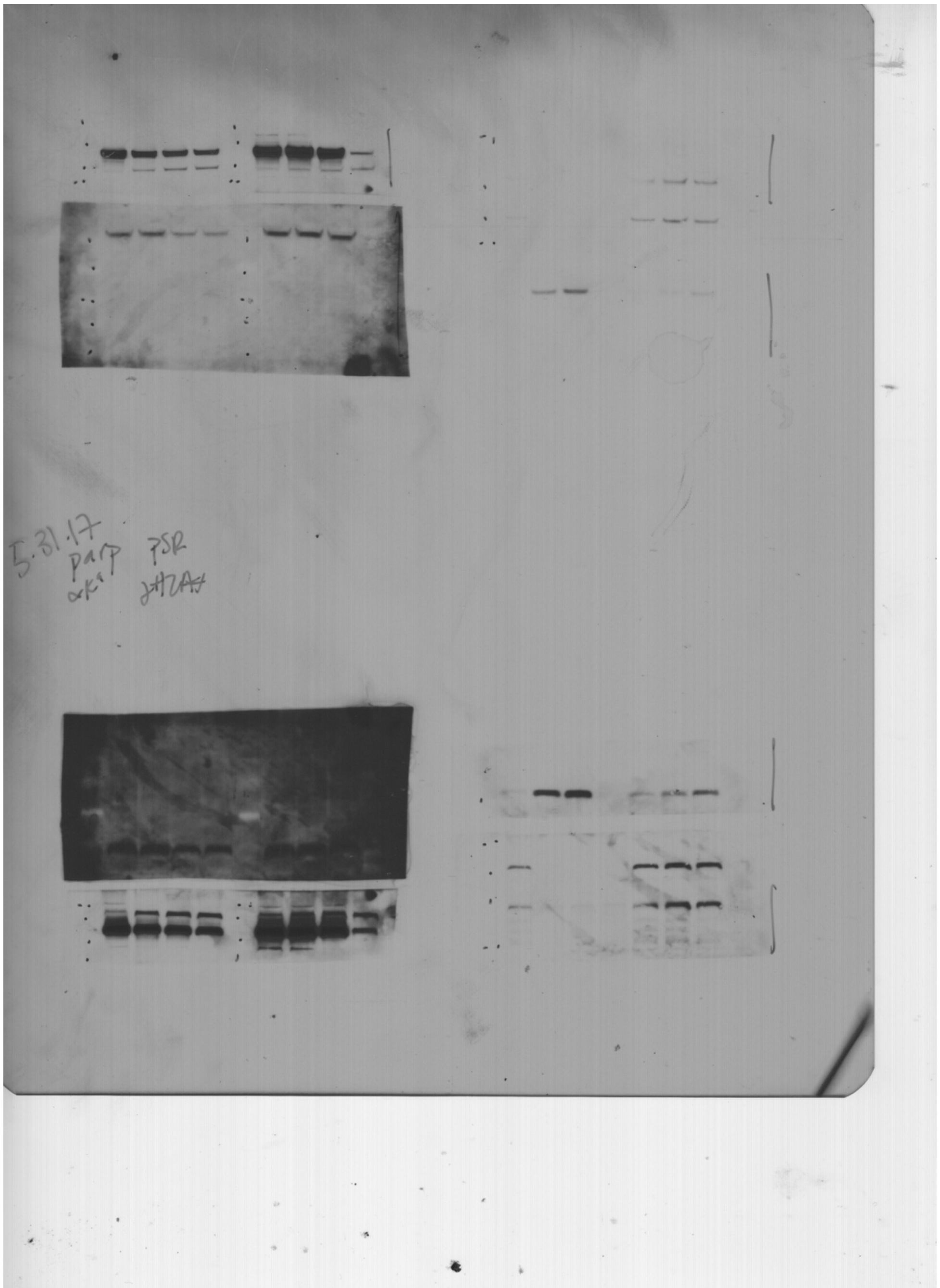

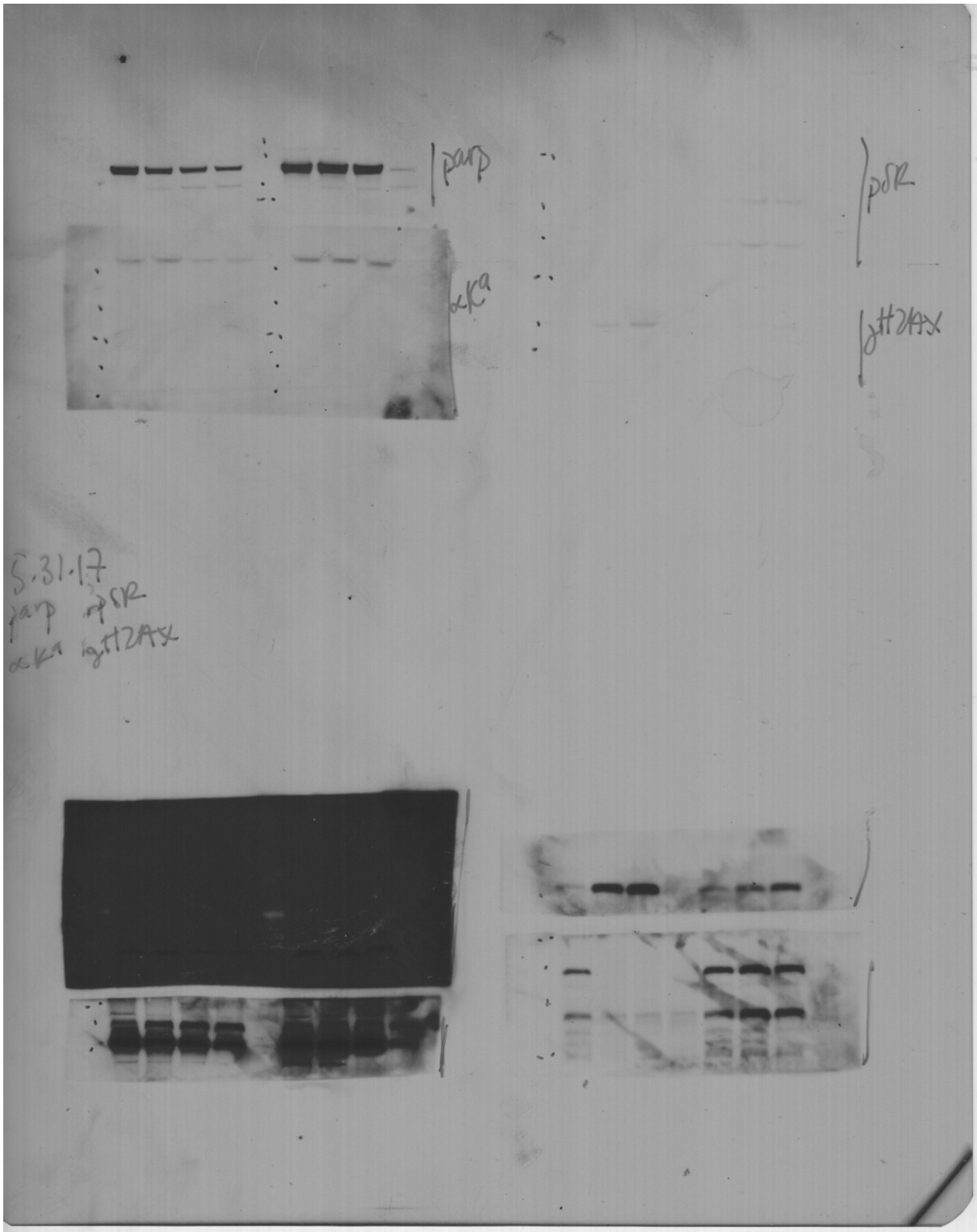
parp
5.31.17

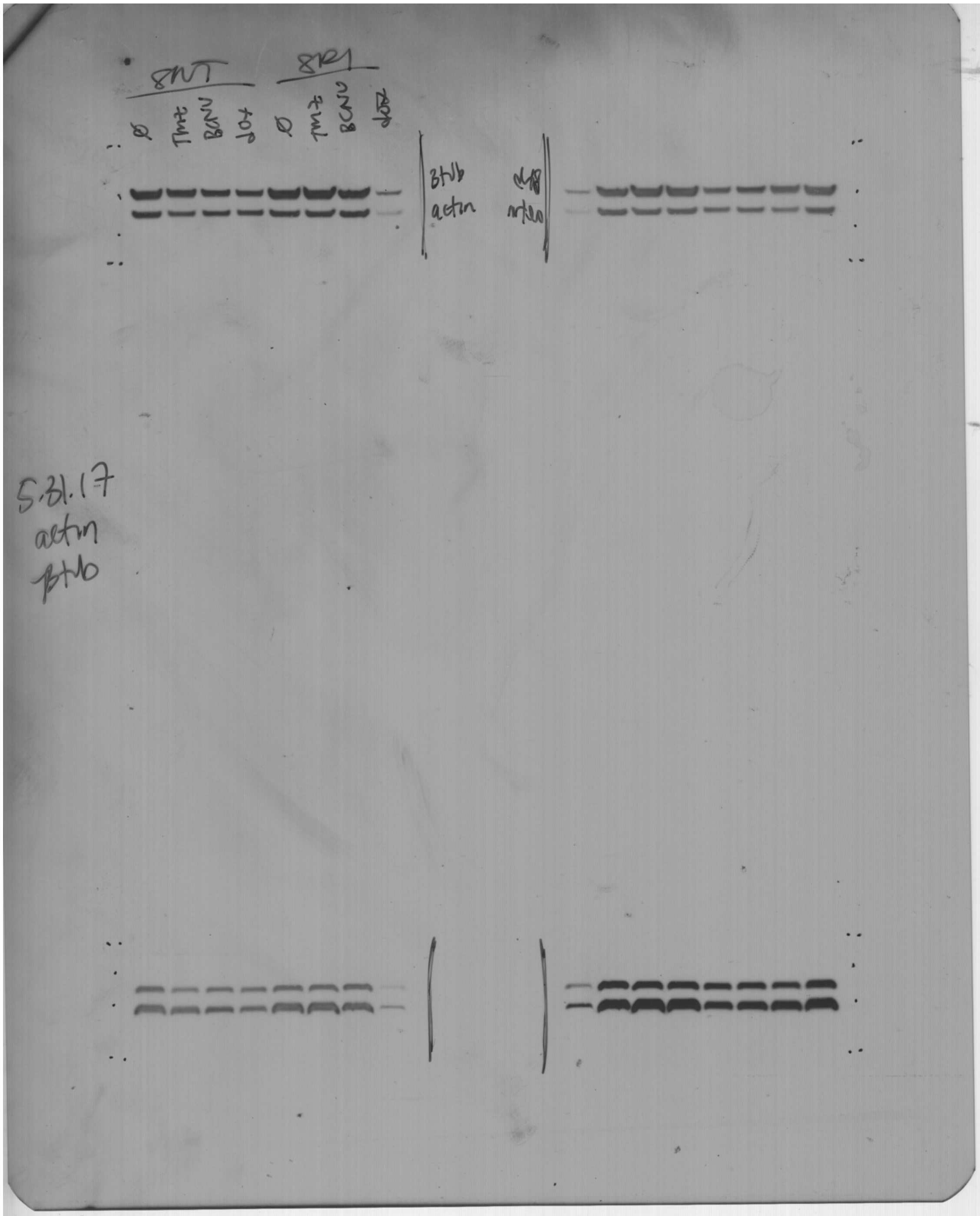
8WT
8R1
Ø
Tmz
BCNU
Ø
Tmz
BCNU
βtub
actin
5.31.17
actin
βtub

Figure 1g full blots. Lanes are: 42MGBA-WT(DMSO, TMZ, BCNU) 42MGBA-TMZres(DMSO, TMZ, BCNU). Antibodies on the right are not relevant to this paper.

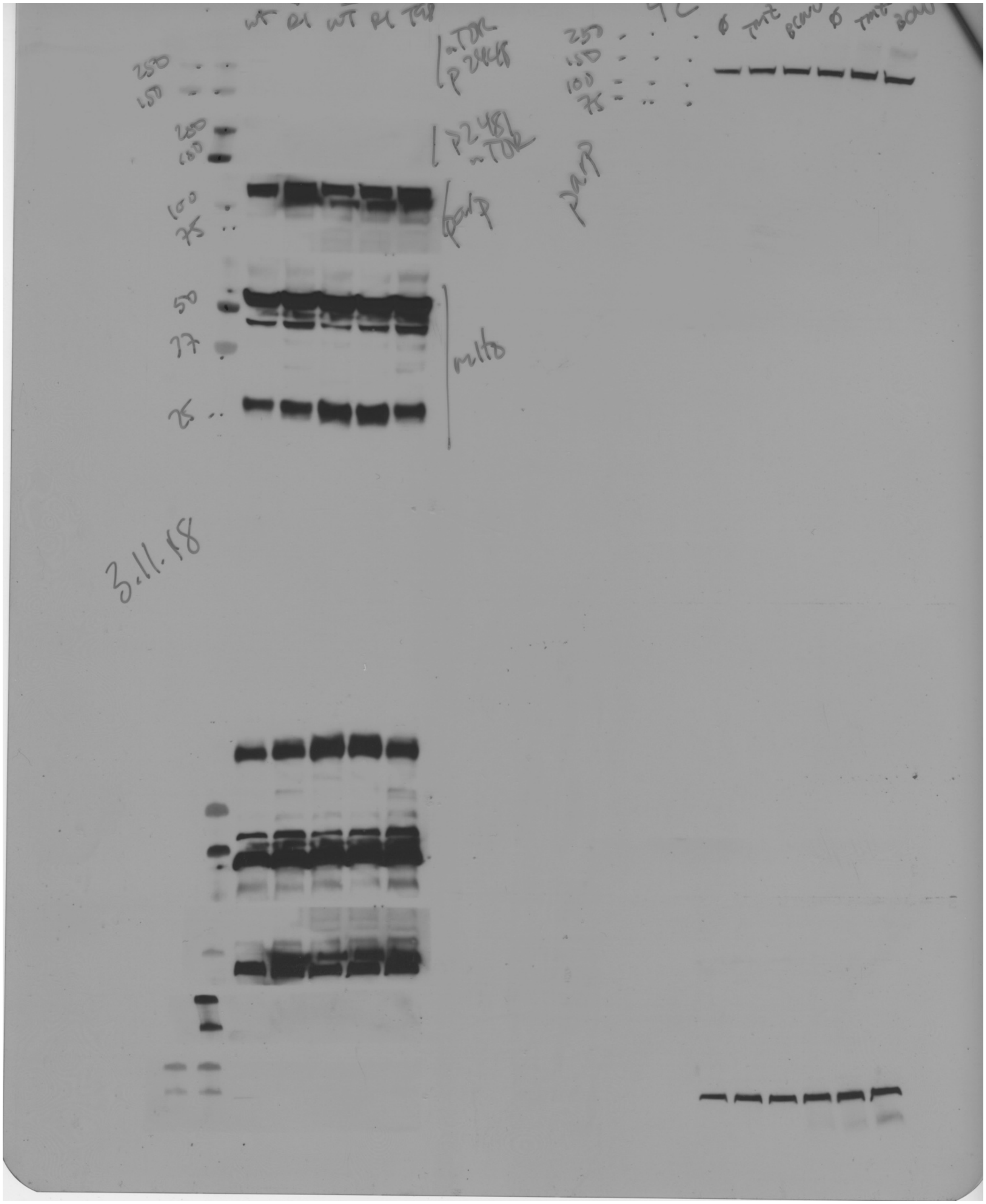

Loading control for 42MBGA-WT and -TMZres DMSO, TMZ, BCNU treated blot for PARP expression.

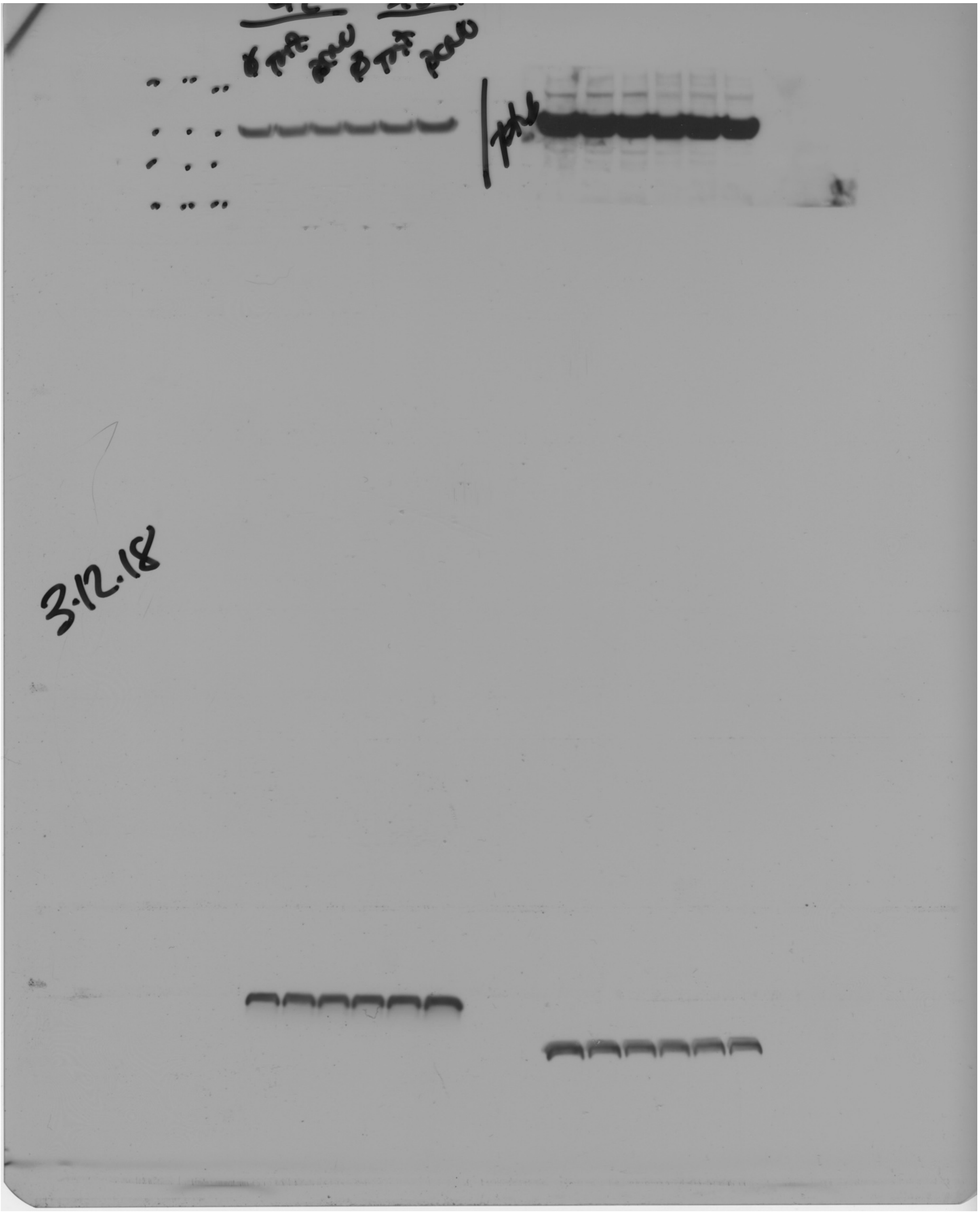

Figure 6d Electron Transport Chain cocktail antibody full blots. Cell lines are 42MBGA-WT, 42MBGA-TMZres, 8MBGA-WT, 8MBGA-TMZres, and T98G. T98G was cropped for this paper as it is not relevant to these studies. As the abundance or affinity of the antibodies in the cocktail differed, two exposure times were used to accurately capture the expression of the mitochondrial electron transport chain complexes.

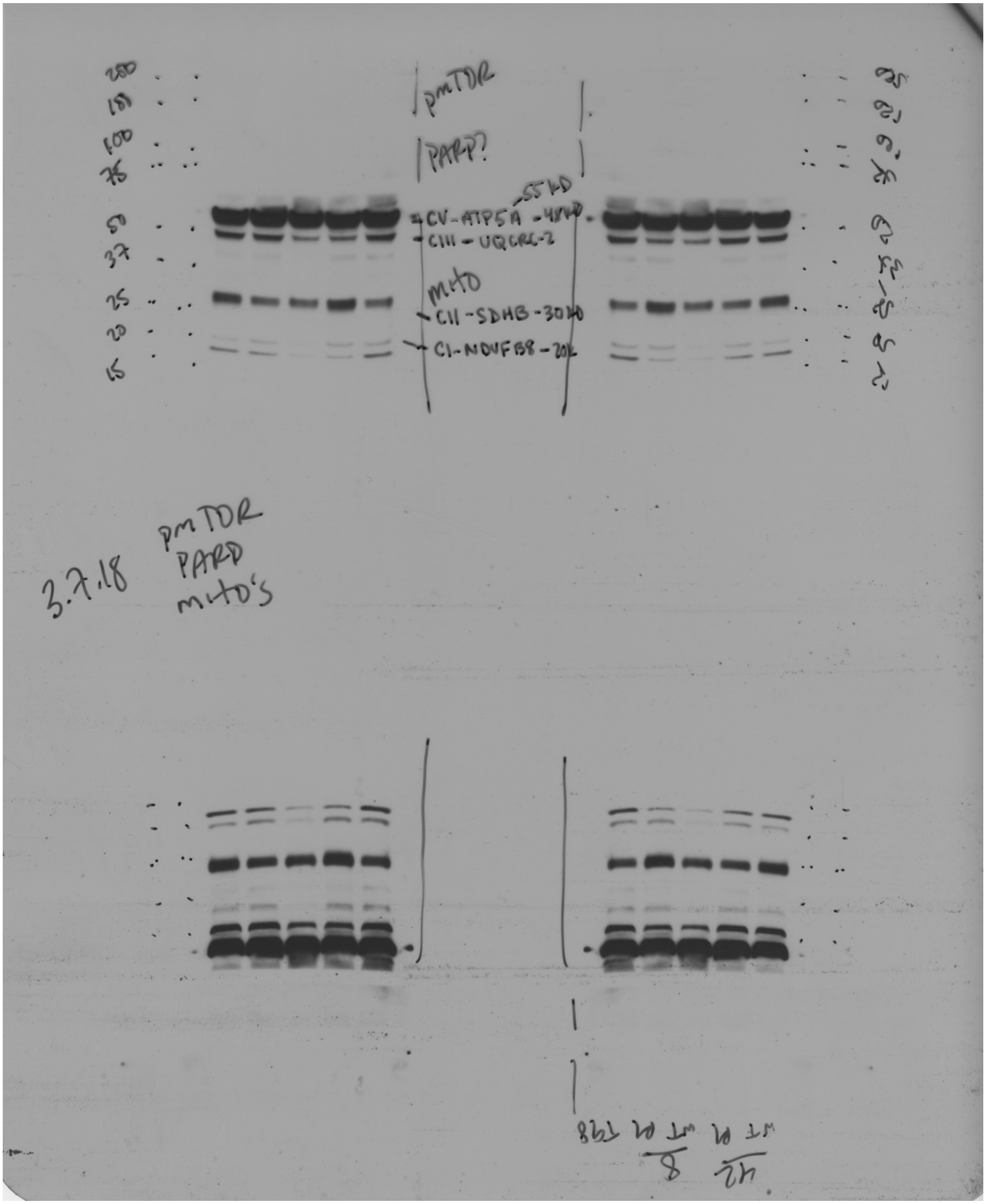

Longer exposure:

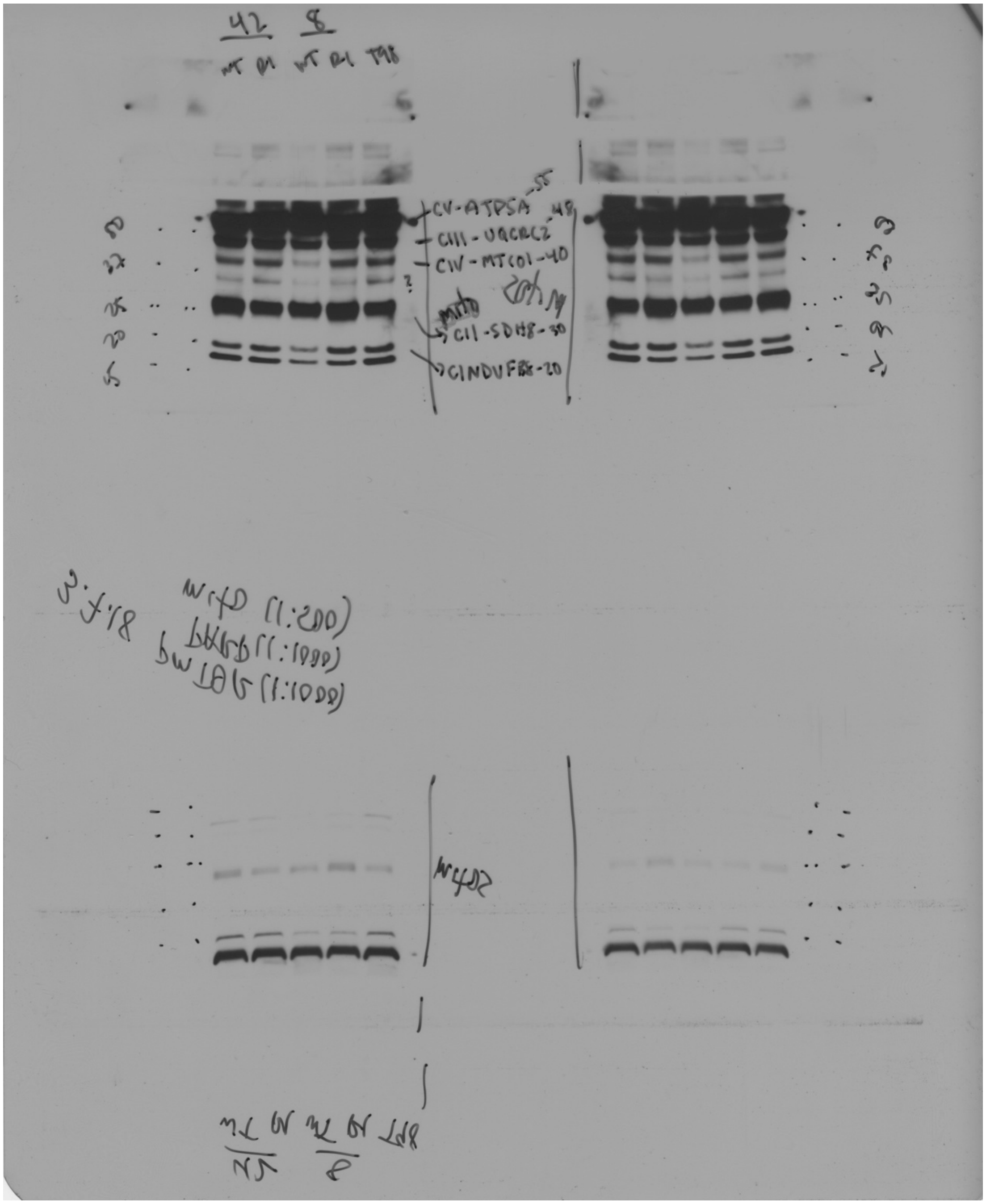

Beta-tubulin loading control for Mitochondrial ETC antibody.

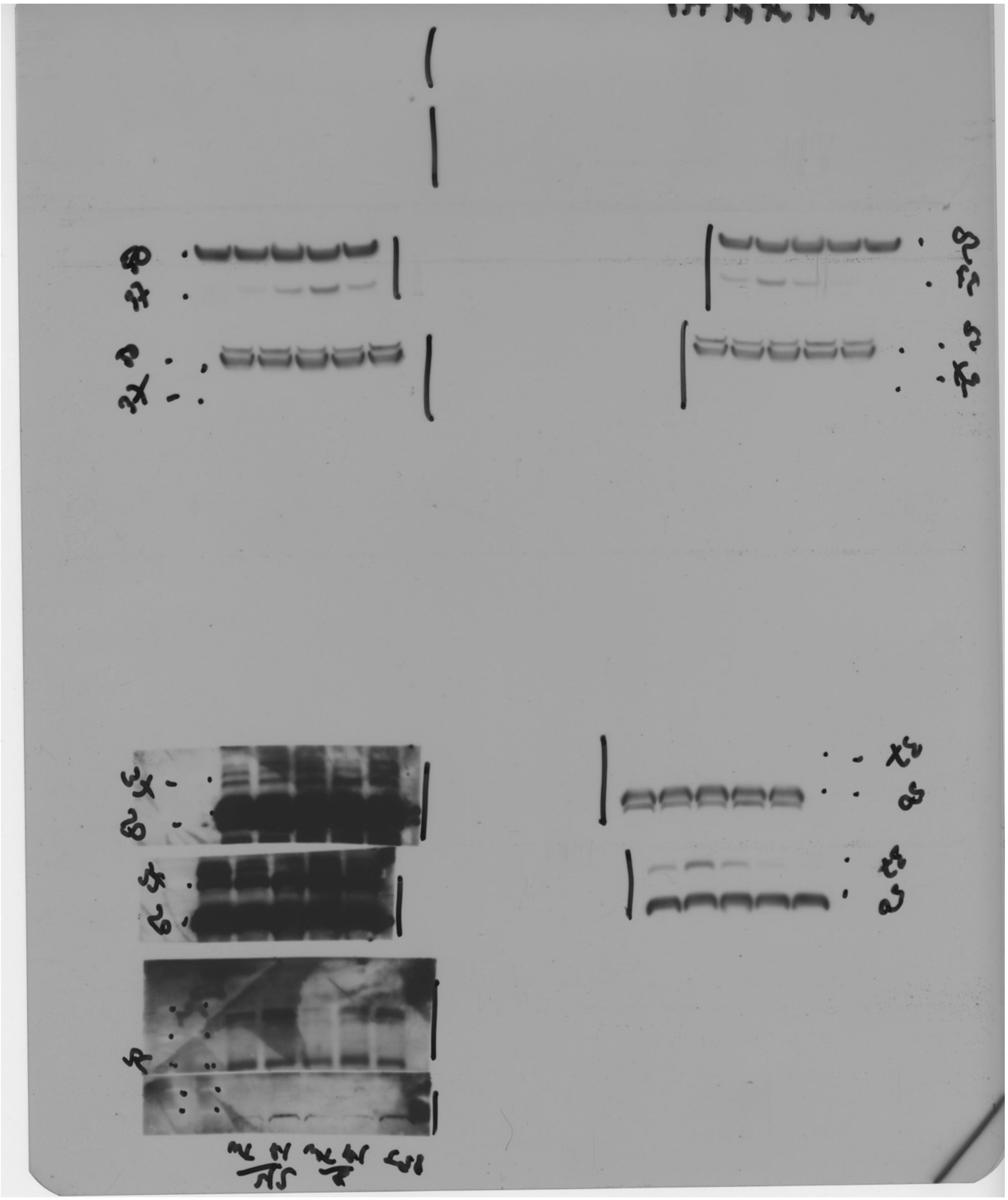

Supplement: Supplementary file 1 — Supplementary Figures [file 41598_2018_25588_MOESM1_ESM.pdf]
